# Multi-scale Fisher’s independence test for multivariate dependence

**DOI:** 10.1093/biomet/asac013

**Published:** 2022-02-21

**Authors:** S. GORSKY, L. MA

**Affiliations:** Department of Mathematics and Statistics, University of Massachusetts Amherst, 710 N. Pleasant Street, Amherst, Massachusetts 01003, U.S.A.; Department of Statistical Science, Duke University, Box 90251, Durham, North Carolina 27708, U.S.A.

**Keywords:** Massive data, Multiple testing, Nonparametric inference, Scalable inference, Unsupervised learning

## Abstract

Identifying dependency in multivariate data is a common inference task that arises in numerous applications. However, existing nonparametric independence tests typically require computation that scales at least quadratically with the sample size, making it difficult to apply them in the presence of massive sample sizes. Moreover, resampling is usually necessary to evaluate the statistical significance of the resulting test statistics at finite sample sizes, further worsening the computational burden. We introduce a scalable, resampling-free approach to testing the independence between two random vectors by breaking down the task into simple univariate tests of independence on a collection of 2 × 2 contingency tables constructed through sequential coarse-to-fine discretization of the sample space, transforming the inference task into a multiple testing problem that can be completed with almost linear complexity with respect to the sample size. To address increasing dimensionality, we introduce a coarse-to-fine sequential adaptive procedure that exploits the spatial features of dependency structures. We derive a finite-sample theory that guarantees the inferential validity of our adaptive procedure at any given sample size. We show that our approach can achieve strong control of the level of the testing procedure at any sample size without resampling or asymptotic approximation and establish its large-sample consistency. We demonstrate through an extensive simulation study its substantial computational advantage in comparison to existing approaches while achieving robust statistical power under various dependency scenarios, and illustrate how its divide-and-conquer nature can be exploited to not just test independence, but to learn the nature of the underlying dependency. Finally, we demonstrate the use of our method through analysing a dataset from a flow cytometry experiment.

## 1. Introduction

Testing independence and learning the dependency structure in multivariate problems has been a central inference task since the very beginning of modern statistics, and the last two decades have witnessed a surge of interest in this problem among statisticians, engineers and computer scientists. A variety of different methods have been proposed for testing independence between two random vectors. For example, Székely & Rizzo (2009) generalized the product-moment covariance and correlation to the distance covariance and correlation. Bakirov et al. (2006), Meintanis & Iliopoulos (2008) and Fan et al. (2017) all developed nonparametric tests of independence based on the distance between the empirical joint characteristic function of the random vectors and the product of the marginal empirical characteristic functions of the two random vectors. Székely & Rizzo (2013) further considered an asymptotic scenario with the dimensionality of the vectors increasing to infinity while keeping the sample size fixed. In a different vein, Heller et al. (2013) formed a test based on univariate tests of independence between the distances of each of the random vectors from a central point. In machine learning, a class of kernel-based tests has also become popular. For example, Gretton et al. (2008) formed a test based on the eigenspectrum of covariance operators in a reproducing kernel Hilbert space. More recently, Pfister et al. (2018) generalized this approach to the multivariate case by embedding the joint distribution into a reproducing kernel Hilbert space. Weihs et al. (2018) defined a class of multivariate nonparametric measures that leads to multivariate extensions of the Bergsma–Dassios sign covariance. Lee et al. (2021) proposed using random projections to reduce multivariate independence testing to a univariate problem, and completed the latter using an ensemble approach combining the distance correlation and a binary expansion test statistic (Zhang, 2019). Berrett et al. (2021) developed a *U*-statistic-based permutation test. Shi et al. (2022) combined the distance covariance with the centre-outward ranks and signs to create a nonparametric test.

Beyond the task of testing, many authors have studied more generally the quantification and estimation of multivariate dependence. For example, Deb et al. (2020) recently proposed a new dependency measure for random variables on general topological spaces. In Sen & Deb (2022) a notion of multivariate rank is defined using the theory of measure transportation, and a multivariate rank version of the distance covariance is proposed. Other classical approaches include graph-based methods (Friedman & Rafsky, 1983; Berrett & Samworth, 2019; Azadkia & Chatterjee, 2021) and copula-based methods (Jaworski et al., 2010).

With some recent exceptions, such as Deb et al. (2020), the existing multivariate independence tests generally require the computation of statistics at a computational complexity that scales at least quadratically in the sample size, making them impractical for datasets with sample sizes greater than, say, tens of thousands of observations. Many of these multivariate methods also require resampling, in the form of either permutation or bootstrap, to evaluate statistical significance. This additional computational burden makes applications of these methods computationally expensive even for problems with moderate sample sizes.To overcome these challenges, some appeal to asymptotic approximations, either in large *n* or in large *p* (Székely & Rizzo, 2013; Pfister et al., 2018), to derive procedures that, when the asymptotic conditions are satisfied, do not require resampling. However, because it is hard to judge whether such conditions are true in multivariate settings, practitioners usually still resort to resampling to ensure validity.

A scalable testing strategy for data with massive sample sizes should ideally achieve (i) close to linear computational complexity in the sample size and (ii) finite-sample guarantees without the need for resampling or asymptotic approximation. We aim to introduce a framework that achieves these two desiderata. Specifically, instead of calculating a single test statistic for independence all at once, we take a multi-scale divide-and-conquer approach that breaks apart the nonparametric multivariate test of independence into simple univariate independence tests on a collection of 2 × 2 contingency tables defined by sequentially discretizing the original sample space at a cascade of scales. This approach transforms a complex nonparametric testing problem into a multiple testing problem involving simple tests that can be carried out efficiently. While such an approach was previously adopted by Ma & Mao (2019) for testing the independence between two scalar variables, the increasing dimensionality in the multivariate setting makes a brute-force, exhaustive approach as proposed therein computationally prohibitive and statistically inefficient. As such, we incorporate data adaptivity into the framework and introduce a coarse-to-fine sequential adaptive testing procedure that exploits the spatial characteristics of dependency structures to drastically reduce the number of univariate tests completed in the procedure. At the same time, we derive a finite-sample theory showing that, even with the additional adaptivity, exact inference, in terms of controlling the level of the test, can be achieved at any given sample size without resorting to either resampling or large-sample approximation. While the main focus of our paper is not on the asymptotic properties, we do also establish the asymptotic consistency of our method under suitable conditions.

Aside from these statistical and computational considerations, our approach also enjoys a feature highly relevant in practical applications. That is, its divide-and-conquer nature allows learning the structure of the underlying dependency. In many modern applications, not only is the practitioner interested in testing the presence of dependency, but usually in also understanding the nature of such dependency. By identifying and visualizing the 2 × 2 tables on which the univariate independence test returns the most significant *p*-values, we can identify interesting dependency relationships otherwise hidden by the multivariate nature of the sample space and the complexity of the joint distribution. Lee et al. (2021) also aimed to learn the nature of the dependency through a multi-scale approach.

We carry out extensive simulation studies that examine the computational scalability and statistical power of our method in a variety of dependency scenarios and compare our method to a number of state-of-the-art approaches. We demonstrate an application of our method to a dataset from a flow cytometry experiment with a massive sample size.

## 2. Method

### 2.1. Multi-scale 2 × 2 testing for multivariate independence

We start by introducing some notation that will be used throughout the paper as well as some concepts related to nested dyadic partitioning, which will be used for constructing the 2 × 2 tables on which univariate independence tests are completed.

Let Ω = Ω_*X*_ × Ω_*Y*_ denote a *D*-dimensional joint sample space of two random vectors *X* and *Y*, where Ω_*X*_ and Ω_*Y*_ are respectively the marginal sample spaces of *X* and *Y*. For simplicity, we assume that $\Omega_{X}={\left[0,1\right]}^{D_{x}}$ and $\Omega_{Y}={\left[0,1\right]}^{D_{y}}$, that is, each marginal random variable of the two random vectors is supported on [0, 1]. This costs no generality as other random variables can be mapped onto the unit interval through, for example, a cumulative distribution function transform.

A partition P on a set *S* is a collection of disjoint nonempty subsets of *S* whose union is *S*. A nested dyadic partition on *S* is a sequence of partitions, $P^{0}$, $P^{1}$, …, $P^{\kappa }$, …, such that $P^{0}=\left\{S\right\}$, and for each *κ* ⩾ 1, the sets in $P^{\kappa }$ are those generated by dividing each set in $P^{\kappa -1}$ into two children. For example, if we consider a nested dyadic partition on [0, 1] generated from sequentially dividing sets into two halves in the middle of the interval, then we have a nested dyadic partition such that, for $\kappa ⩾0,P^{\kappa }={\left\{\left[(l-1)/2^{\kappa },l/2^{\kappa }\right)\right\}}_{l\in \left\{1,\ldots ,2^{\kappa }\right\}}$. We refer to this particular nested dyadic partition as the canonical nested dyadic partition, and note that $\cup P^{\kappa }$ generates the Borel *σ*-algebra. In the following, we consider only nested dyadic partitions that generate the Borel *σ*-algebra. Now let us assume that each dimension of Ω has a corresponding nested dyadic partition. For our purpose, the nested dyadic partition for each dimension can be distinct, but for ease of illustration, let us assume that they are all the canonical nested dyadic partitions on [0, 1]. We consider the cross-products of these marginal nested dyadic partitions on each dimension, which creates a cascade of partitions on the joint sample space. Specifically, for any vector of nonnegative integers $k=\left(k_{1},\ldots ,k_{D}\right)\in {\mathbb{N}}_{0}^{D},P^{k_{1}}\times \cdots \times P^{k_{D}}$ forms a partition of Ω. The elements of this partition are rectangles of the form

$$A=A_{1}\times A_{2}\times \cdots \times A_{D}\text{ with }A_{d}\in P^{k_{d}}\text{ for all }d=1,2,\ldots ,D.$$

**Fig. 1.**
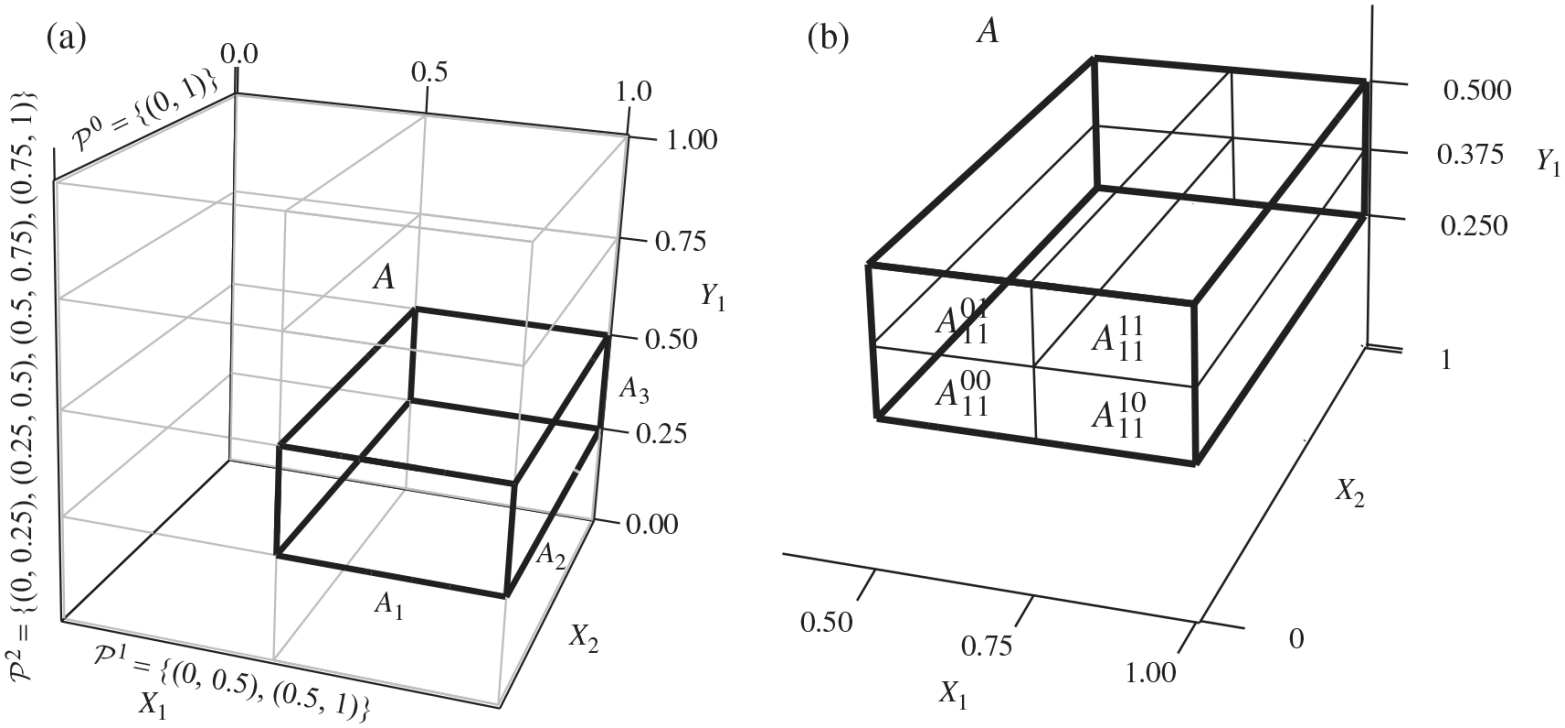
(a) A cuboid of resolution 3 in a three-dimensional sample space under the canonical nested dyadic partition. (b) The division of the cuboid *A* in (a) into four blocks along dimension 1 for *X* and dimension 1 for *Y*.

The vector *k* encodes the level of *A* in the nested dyadic partition for each dimension of Ω. That is, *k*_*d*_ is the level of the nested dyadic partition on [0, 1] to which the *d*th margin of *A* belongs. From now on, we refer to a set *A* of the above form as a cuboid. We refer to the sum *r* = *k*_1_ + ··· + *k*_*D*_ as the resolution of *A*. Figure 1(a) illustrates a cuboid *A* of resolution 3 in a three-dimensional sample space with canonical nested dyadic partitions on the margins.

We are now ready to construct the 2 × 2 tables on which to carry out univariate tests of independence. One can divide a cuboid *A* into four blocks according to the nested dyadic partition along any pair of its margins while keeping all other dimensions intact. For the division involving dimension *i* of *X* and dimension *j* of *Y*, we use $A_{ij}^{00}$, $A_{ij}^{01}$, $A_{ij}^{10}$ and $A_{ij}^{11}$ to denote these four blocks. Figure 1(b) illustrates a division on the cuboid demonstrated in Fig. 1(a).

Suppose now that *F* is the joint sampling distribution of (*X*, *Y*); then, for the 2 × 2 division of *A* along the *i*th dimension of *X* and *j*th dimension of *Y*, we can define a corresponding odds ratio that characterizes the dependency in *F* on the 2 × 2 division,

$$\theta_{ij}(A)=\frac{F\left(A_{ij}^{10}\right)F\left(A_{ij}^{01}\right)}{F\left(A_{ij}^{00}\right)F\left(A_{ij}^{11}\right)}.$$

An independent and identically distributed sample from *F* will give rise to a 2 × 2 contingency table formed by the number of data points lying in the four blocks 

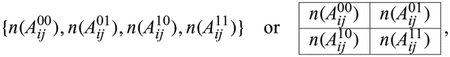

 where *n*(*A*) represents the number of data points in *A*.

**Fig. 2.**
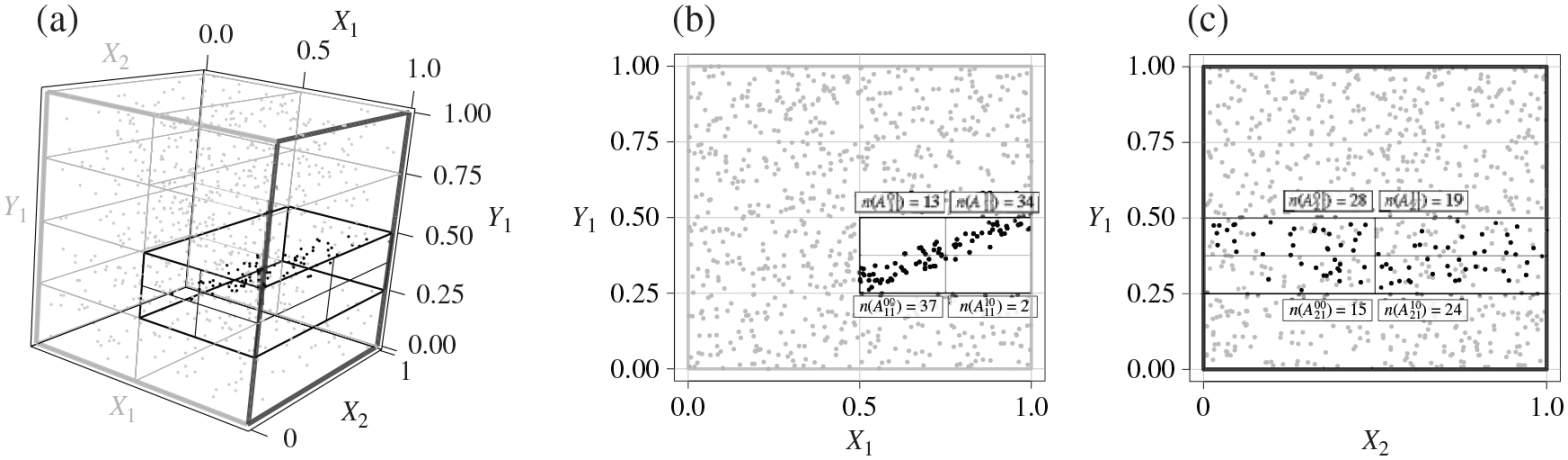
Illustration of the two 2 × 2 contingency tables on a cuboid *A* arising from an independent and identically distributed sample in which dependency exists in (*X*_1_, *Y*_1_).

One can test whether *θ*_*ij*_(*A*) = 1 based on this contingency table. While several standard tests are available for testing independence on a 2 × 2 table, we adopt Fisher’s exact test. As we will show in § 2.3, it turns out that the conditional nature of Fisher’s test plays a crucial role in our finite-sample theory; it ensures that the resulting testing procedure obtains exact validity at any finite sample size without resampling or asymptotics. Figure 2 illustrates two contingency tables on which Fisher’s test is applied for a cuboid *A*. In the following, we use *p*_*ij*_(*A*) to represent the resulting *p*-value from the test on this particular 2 × 2 table.

How does testing those local nulls *θ*_*ij*_(*A*) = 1 relate to our original global hypothesis of *X* ⫫ *Y*? It is obvious that if *X* ⫫ *Y* then independence must hold, that is, *θ*_*ij*_(*A*) = 1 for any *A* and any pair of *X*-*Y* margins *i* and *j*. However, the reverse is not obvious. Does independence on these 2 × 2 tables formed under the marginal nested dyadic partitions also imply that *X* and *Y* are independent? If this is the case then one can test for independence between *X* and *Y* by testing whether *θ*_*ij*_(*A*) = 1 on the 2 × 2 tables. The next theorem confirms that this is indeed the case.

Theorem 1. *We have X* ⫫ *Y if and only if θ*_*ij*_(*A*) = 1 *for all pairs of dimension i of X and dimension j of Y on all cuboids A*.

This theorem implies that one can in principle test for independence between two random vectors *X* and *Y* by exhaustively testing whether independence holds on each of the 2 × 2 tables constructed on all cuboids up to some maximum resolution, aimed at identifying dependency structures up to a certain level of detail. This boils down to a multiple testing problem involving a collection of *p*-values computed on all of the 2 × 2 tables up to the maximal resolution. However, such a brute-force exhaustive scan is not practical when the dimensionality grows. If one were to exhaustively test independence on all possible 2 × 2 tables of all cuboids up to even just a moderate resolution, the number of tests required would quickly become prohibitive. Specifically, the total number of tests to be completed up to a resolution of *R* is

$$\overset{R}{\underset{\rho =0}{\sum }}D_{x}D_{y}2^{\rho }\left(\begin{array}{c} \rho +D-1\\ D-1 \end{array}\right).$$

For multivariate problems of more than a handful of dimensions, one must be selective in carrying out the univariate tests. Beyond the consideration of computational practicality, reducing the number of tests is also desirable for the sake of statistical performance. Every additional test comes with a price in multiple testing control, and thus it is important to be discreet in choosing the tests to complete.

### 2.2. Multi-scale Fisher’s independence test: a coarse-to-fine adaptive testing procedure

**Fig. 3.**
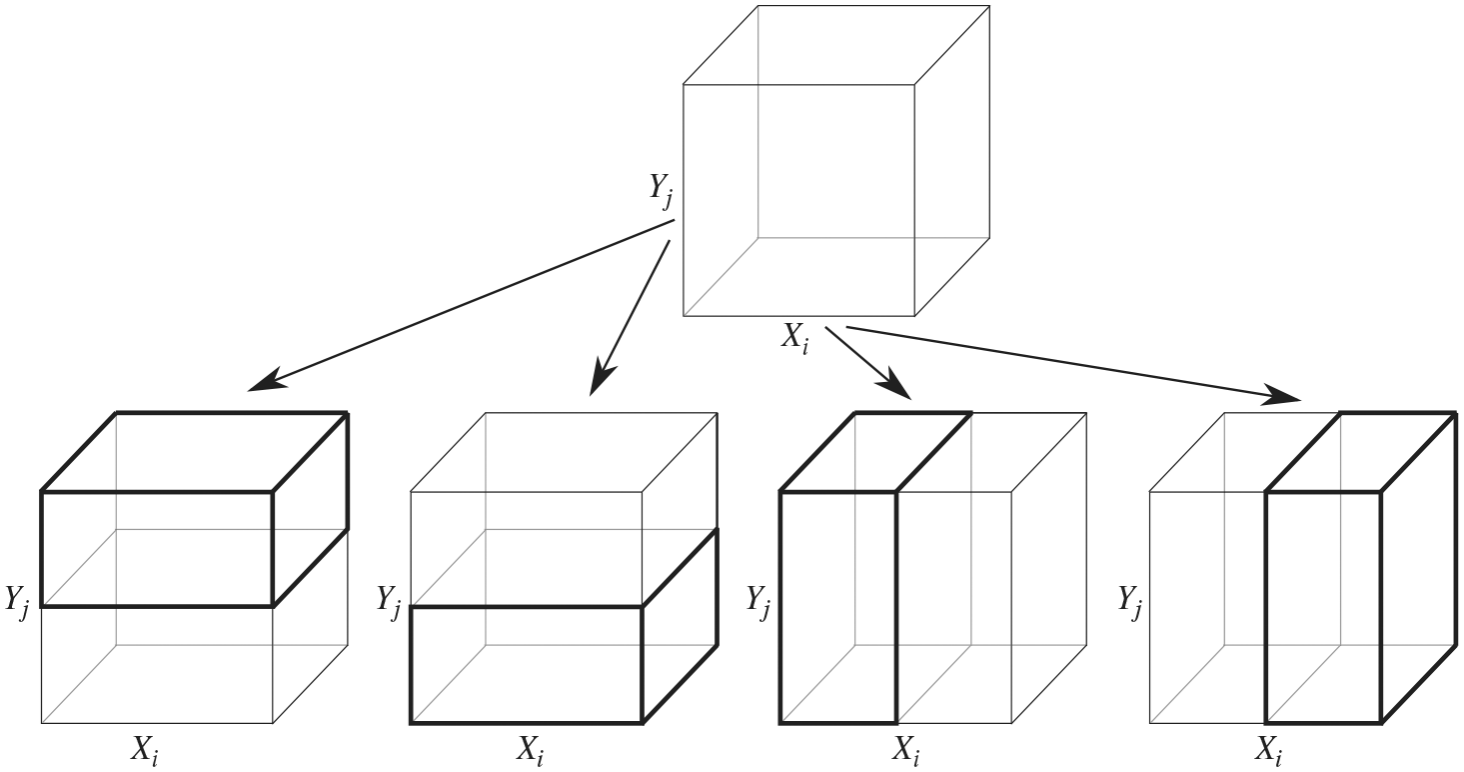
The selection of tables for testing based on the statistical evidence on their parent. The two right children correspond to dividing *A* along the *i*th margin of *X*, and the two left children correspond to dividing *A* along the *j*th margin of *Y*. Those four children are tested in resolution *r*+1 if their parent *A* in resolution *r* produces a *p*-value, *p*_*ij*_(*A*), below the threshold p*.

Given the above considerations, we propose a data-adaptive strategy that selects in each resolution a subset of the available tables to test based on the statistical evidence attained on coarser resolutions. In particular, only the children of tables in the previous resolution whose *p*-values are below a prespecified threshold are selected for testing. Figure 3 provides an illustration. Suppose that cuboid *A* in resolution *r* satisfies *p*_*ij*_(*A*) < p*, some preset threshold; then the four children cuboids, generated by dividing *A* in the *i*th or the *j*th dimension, are tested in resolution *r*+1. This coarse-to-fine testing procedure terminates at a maximal resolution *R*_max_, or when no cuboids at the current resolution have *p*-values passing the threshold.

The rationale behind this strategy is to exploit the spatial smoothness of dependency structures. When *X* and *Y* are dependent, adjacent and nested cuboids tend to contain empirical evidence for the dependency in a correlated manner. Here the correlation corresponds to our assumption about the underlying sampling distribution that its dependency structure is spatially smooth, not the sampling behaviour of the data points given the sampling distribution. Thus, using the statistical evidence at coarser resolutions to inform which cuboids to test in finer resolutions can lead to effective detection of the dependency structure.

Next we formally present the adaptive testing procedure that we call MultiFIT, which stands for multi-scale Fisher’s independence test. We let $C^{\left(r\right)}$ denote the collection of cuboids at resolution *r* on which we carry out independence tests over all of the corresponding *D*_*x*_*D*_*y*_ 2 × 2 tables, one for each (*i*, *j*) pair of margins, where *i* and *j* are the indices for the *X* and *Y* margins, respectively. The MultiFIT procedure consists of three main steps.

*Step 0*. Initialization: let $C^{\left(0\right)}$ be {Ω}, and let $C^{\left(r\right)}=\emptyset$ for 1 ⩽ *r* ⩽ *R*_max_.

*Step 1*. Coarse-to-fine scanning: for *r* = 0 to *r* = *R*_max_, do the following.

- Independence testing: apply Fisher’s exact test of independence to the *D*_*x*_*D*_*y*_ 2 × 2 tables of each cuboid $A\in C^{\left(r\right)}$ and record the *p*-values.
- Selection of cuboids to test for the next resolution: when *r* < *R*_max_, if the (*i*, *j*) table for a cuboid $A\in C^{\left(r\right)}$ has a *p*-value more significant than a threshold p*, add to $C^{\left(r+1\right)}$ the four child cuboids of *A* generated from dividing *A* along the *i*th and the *j*th dimensions of *X* and *Y*, respectively, each generating two children.

*Step 2*. Multiple testing control: apply any valid multiple testing control procedure on the entire set of *p*-values generated by the algorithm, thereby controlling the level of the entire testing procedure at *α*.

Although the data-adaptive selection in Step 1(b) is designed to overcome the explosive number of tests required when the dimensionality is large, it is still often feasible to apply exhaustive testing up to some resolution R* < *R*_max_. In other words, one can test on all available cuboids up to resolution R*, and let the adaptive selection of the cuboids in Step 1(b) kick in for resolutions beyond R*. In our software, we allow the user to specify a resolution R* below which exhaustive testing is adopted. A smaller value for R* will favour the detection of more global signals, while a larger R* will favour localized signals.

In our implementation of the testing procedure, we consider two different approaches for achieving the multiple testing control in Step 2.

#### Strategy 1 (A holistic approach to multiple testing).

Under this strategy, one applies multiple testing control on the entire set of *p*-values generated in Step 1 of the MultiFIT procedure all at once, regardless of the resolution of the corresponding table. Simple choices of the multiple testing devices include Bonferroni and Holm corrections.

#### Strategy 2 (A resolution-specific approach to multiple testing).

Under this strategy, one applies multiple testing control in two stages: first on the *p*-values within each resolution level, producing an intermediate, intraresolution significance level for each resolution; and then in the second stage further correct these intraresolution *p*-values over all the resolutions, which will produce a valid, corrected overall *p*-value for testing the global null hypothesis of independence. This strategy has the benefit that one can now allocate a fixed level budget to each resolution, and thus avoids the possibility of loss in power due to having many more tables tested in high resolutions than coarse ones. This method is generally more powerful than the holistic approach for testing the global null hypothesis when a dependency structure exists in coarser resolutions.

An additional benefit of the resolution-specific approach is that it can be implemented with early stopping so that the MultiFIT procedure can terminate as soon as there is sufficient evidence for rejecting the global null in the first few resolutions without continuing into testing on higher resolutions. This is possible because in this approach we bound the influence of tables in finer resolutions on the corrected significance level of tests in coarser resolutions. Our software implements this early stopping strategy for the resolution-specific approach to multiple testing, when Holm’s method is used for intraresolution correction along with Bonferroni’s method for cross-resolution correction. Early stopping can reduce the time complexity significantly in the presence of a global signal.

### 2.3. Finite-sample validity and large-sample consistency

Because the multi-scale Fisher’s independence test formulates the test of independence as a multiple testing problem, its inferential validity rests on whether the *p*-values are indeed valid, i.e., that they are stochastically larger than a uniform random variable under the null hypothesis. The *p*-values for the cuboids selected in the MultiFIT procedure are computed according to the central hypergeometric null distribution on the 2 × 2 tables. At first glance, these null distributions appear to ignore the data-adaptive selection of a cuboid *A* based on the evidence in its ancestral cuboids. As such, one may suspect that there might be a selection bias that causes such *p*-values to lose their face values.

The following theorem and corollary resolve this concern by showing that, interestingly, the distribution of all the selected 2 × 2 tables, given their marginal totals are independent of the event that they are selected in the procedure, and hence the *p*-values computed in the procedure are indeed still valid despite the adaptive sequential selection. Consequently, one can indeed control the level of the entire procedure using multiple testing methods based on these *p*-values.

Theorem 2. *Under the null hypothesis X* ⫫ *Y*,

$$n\left(A_{ij}^{00}\right)⫫I\left(A\in C^{\left(r\right)}\right)|n\left(A_{ij}^{0\cdot }\right),n\left(A_{ij}^{\cdot 0}\right),n(A)$$

*for all cuboids A of resolution r and all pairs* (*i*, *j*) *of the margins, where $n\left(A_{ij}^{0\cdot }\right)=n\left(A_{ij}^{00}\right)+n\left(A_{ij}^{01}\right)$ and $n\left(A_{ij}^{\cdot 0}\right)=n\left(A_{ij}^{00}\right)+n\left(A_{ij}^{10}\right)$*, *and $I\left(A\in C^{\left(r\right)}\right)$ is the indicator for the event that A is selected to be tested in the* MultiFIT *procedure*.

In other words, for any cuboid *A*, the conditional distribution of the 2 × 2 table on each pair of *X*-*Y* margins, given the corresponding marginal totals is the same central hypergeometric distribution when *X* ⫫ *Y*, whether or not we condition on the event that cuboid *A* is selected to be tested in the MultiFIT procedure. As such, the *p*-values from Fisher’s exact tests applied on the adaptively selected tables in our procedure can be treated at face value, which justifies using multiple testing adjustment based on these *p*-values to control the level.

Corollary 1. *The p-values computed during Step 1 of the* MultiFIT *procedure are valid, and thus Step 2 of the procedure can control the level of the entire testing procedure at any given level α*.

The above theorem and corollary provide a strong theoretical guarantee, unavailable to other existing methods, that the multi-scale Fisher’s independence test method attains exact control of the level at any finite sample size. This is an extremely important property in that, for multivariate sample spaces, traditional large *n* asymptotic controls of the level can often be inaccurate, and existing methods typically appeal to resampling strategies such as permutation to provide approximate finite-sample control of the level. But permutation is often computationally prohibitive in this context in that even just a single run of a test can be expensive, not to mention applying the same test hundreds to thousands of times. In contrast, the multi-scale Fisher’s independence test method achieves exact control of the level by a single run of the procedure without resampling. We offer a numerical validation of level control through simulations in § 4.

The proof of Theorem 2 turns out to be conceptually interesting and elucidates why the adoption of Fisher’s exact test on each 2 × 2 table is critical to ensuring the exact finite-sample validity of the multi-scale Fisher’s independence test method. In particular, the event that a cuboid *A* is selected to be tested in the MultiFIT procedure is in the *σ*-algebra generated by the *p*-values on all of its ancestral cuboids, which can be shown to be independent of the counts in the 2 × 2 table on *A* under the null hypothesis of independence once the corresponding marginal totals are conditioned upon. This independence is elucidated under a Bayesian network representation of the multivariate central hypergeometric distribution (Ma & Mao, 2019, Theorem 3). Accordingly, conditioning on the selection of a cuboid under the MultiFIT procedure does not alter the null distribution of the *p*-values for the 2 × 2 tables on that cuboid, and thus the validity of the procedure is maintained even with the adaptive selection of the tables to test on. Another interesting feature of the MultiFIT procedure, which is revealed in the proof of Theorem 2 and follows from the adoption of Fisher’s exact tests on the cuboids, is that the test under the MultiFIT procedure is conditional on the marginal values of *X* and *Y*, and hence remains valid, in terms of level control, even after transforms on the data that are applied marginally to *X* and *Y*, respectively. These transforms, such as the empirical cumulative distribution function or rank transform applied to each margin of the observations, are commonly adopted in practice to enhance the power of existing tests.

Below we provide a sketch of the proof for Theorem 2 for interested readers and defer the technical details to the Supplementary Material.

*Proof of Theorem* 2. For two nonnegative integers *a* and *b*, let *n*_*a*,*b*_ denote 2^*a*^ × 2^*b*^ contingency table formed by a cross-product of a marginal partition on *X* at depth *a* and a marginal partition on *Y* at depth *b*. Specifically, it is the 2^*a*^ × 2^*b*^ contingency table corresponding to a partition $P^{k_{1}}\times \cdots \times P^{k_{D}}$ of Ω, where $a=k_{1}+\cdots +k_{D_{x}}$ and $b=k_{D_{x}+1}+\cdots k_{D}$. Under the null hypothesis that *X* ⫫ *Y*, the sampling distribution of any such table *n*_*a*,*b*_ given all of its row totals and column totals is a multivariate central hypergeometric distribution.

By Theorem 3 of Ma & Mao (2019), a draw from the central multivariate hypergeometric distribution such as *n*_*a*,*b*_ can actually be generated inductively from coarse-to-fine resolutions using univariate central hypergeometric distributions. Specifically, suppose that we have already generated the tables *n*_*a*−1,*b*_ and *n*_*a*,*b*−1_; then the conditional distribution of *n*_*a*,*b*_ given its row and column totals, as well as the two parent tables *n*_*a*−1,*b*_ and *n*_*a*,*b*−1_, are simply a collection of independent univariate central hypergeometric distributions, one for each adjacent 2 × 2 subtable in *n*_*a*,*b*_ given its row totals and column totals, which correspond to cell counts in *n*_*a*−1,*b*_ and *n*_*a*,*b*−1_.

**Fig. 4.**
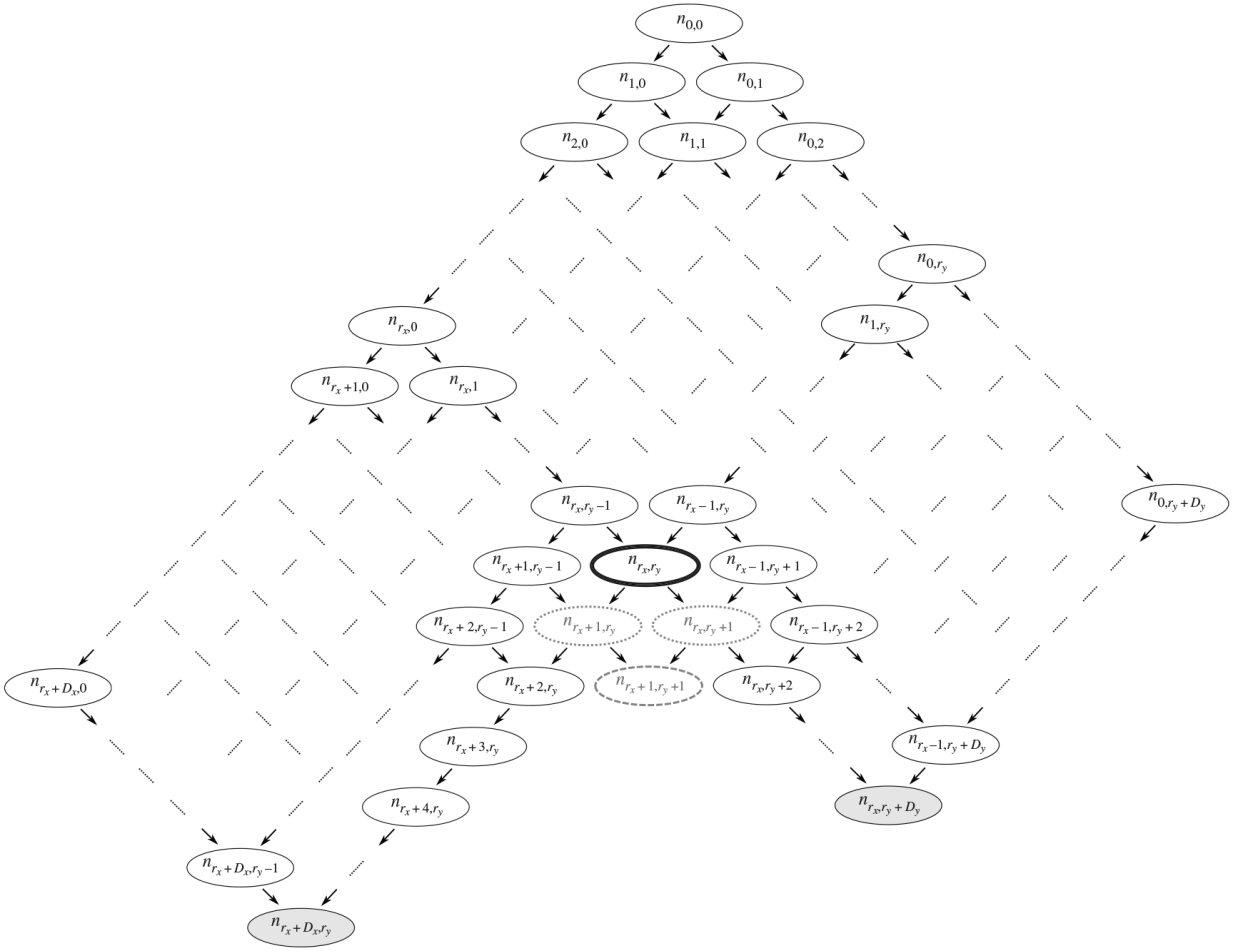
A Bayesian network representation for the multivariate central hypergeometric model on contingency tables formed by cross-products of sequential marginal partitions on Ω_*X*_ and those on Ω_*Y*._

Let *A* be a cuboid that arises from dividing the *X* margins a total of *r*_*x*_ times and the *Y* margins a total of *r*_*y*_ times. The above reasoning implies that one can show by construction that, for any 2 × 2 table on a cuboid *A*, there exists a Bayesian network in the form presented in Fig. 4 such that the total number of observations in *A*, *n*(*A*), is an element in the contingency table $n_{r_{x},r_{y}}$ (the node with the bold black boundary in Fig. 4), the counts for the four blocks of the 2 × 2 table, $n\left(A_{ij}^{00}\right)$, $n\left(A_{ij}^{01}\right)$, $n\left(A_{ij}^{10}\right)$ and $n\left(A_{ij}^{11}\right)$, are in $n_{r_{x}+1,r_{y}+1}$ (the node with the grey dashed boundary in Fig. 4), and the marginal totals of *A* are in $n_{r_{x}+1,r_{y}}$ and $n_{r_{x},r_{y}+1}$ (the two nodes with dotted grey boundaries in Fig. 4). In addition, the counts of all of the 2 × 2 tables on ancestors of *A* are measurable with respect to the *σ*-algebra generated by the grey-shaded nodes in the Bayesian network, and thus are independent of the 2 × 2 table on *A* given the marginal totals. Therefore, the selection of a table does not influence the null distribution once the marginal totals are conditioned upon because such conditioning blocks all the paths from these ancestral nodes to $n_{r_{x}+1,r_{y}+1}$, the node with the grey dashed outline. □

Now that we have established the finite-sample exact validity of the MultiFIT procedure, our last theoretical result shows that, when the sample size *n* grows, under certain conditions, the MultiFIT procedure can consistently reject the null hypothesis of independence.

Theorem 3 (Large-sample consistency). *Suppose that X and Y are not independent under their sampling distribution F. Let* (*X*_1_, *Y*_1_), (*X*_2_, *Y*_2_), …, (*X*_*n*_, *Y*_*n*_) *be independent and identically distributed observations from F. As n* → ∞*, suppose that one of the following conditions holds:*

- R* *is fixed, but large enough such that there exists at least one cuboid A of resolution r* ⩽ R* *with θ*_*ij*_(*A*) ≠ 1 *for some pairs of margin* (*i*, *j*);
- R* → ∞ *and it is o*(log *n*).

*Then the power for the* MultiFIT *procedure to reject the null hypothesis that X* ⫫ *Y converges to* 1.

In practice, conditions (i) and (ii) of Theorem 3 imply that the MultiFIT procedure will perform best when the dependency structure reflects itself in a cuboid at relatively low resolutions. In contrast, the types of dependencies for which the procedure suffers the most substantial power loss are those whose local odds ratios deviate from 1 only in cuboids at high resolutions, while all cuboids at low resolutions have odds ratios equal or close to 1. Such a dependency is highly local in nature and, as will be shown in our simulation analysis, in such cases existing approaches, whose asymptotic conditions generally involve more global properties of the underlying distributions, such as moment conditions, could and often do suffer even more substantial power loss at finite sample sizes in practice.

### 2.4. Practical considerations when applying the MultiFIT procedure

We close this section by discussing some practical aspects in applying the MultiFIT procedure. We set the default value for the *p*-value threshold p* in our software for resolutions higher than R* at {*D*_*x*_*D*_*y*_ log_2_(*n*)}^−1^. This keeps the number of 2 × 2 tables tested constant, on average, under the null hypothesis irrespective of the number of dimensions, while also making the threshold more stringent with increasing sample size in such a way that makes the total number of tables scale roughly linearly with the sample size, which we confirm numerically in the next section.

Under this strategy of setting p*, for certain alternatives, in particular those that are pervasive over the sample space and involve a large number of cuboids, the complexity of the MultiFIT procedure may be higher than *O*(*n* log *n*). Such large-scale, global alternatives, however, can usually be detected in coarse resolutions, and thus in practice, when the algorithm is equipped with early stopping, it will in fact run faster with larger *n* under such alternatives.

If the practitioner wishes to ensure a strict *O*(*n* log *n*) bound on the computational complexity with or without incorporating early stopping, a simple approximate version of the multi-scale Fisher’s independence test algorithm can achieve this. Specifically, in Step 1(b) of the MultiFIT procedure, instead of including child cuboids of all cuboids with a *p*-value less than p*, one can include the child cuboids of up to a prespecified maximum number of cuboids per resolution with the most significant *p*-values less than p*. This alternative constraint ensures that the computational cost of the MultiFIT procedure is strictly bounded at *O*(*n* log *n*).

While under this approximation the conditions for ensuring the finite-sample guarantees are no longer satisfied, we found in practice that its statistical power, as demonstrated in Figs. 7(a) and 7(b) in § 3.2, and level, as demonstrated in the Supplementary Material, hardly differ from those of the exact MultiFIT procedure in essentially all of the numerical settings we have encountered.

## 3. Numerical examples

### 3.1. Computational scalability

Because computational scalability is a key motivation for our approach, we start by evaluating the computational scalability of the MultiFIT procedure with those of three other state-of-the-art methods with well-documented software, namely, the Heller–Heller–Gorfine multivariate test of association from Heller et al. (2013), the distance covariance method of Székely & Rizzo (2009) and the kernel-based Brownian distance covariance of Pfister et al. (2018).

We apply these methods to datasets simulated under six scenarios described in the Supplementary Material along with a null scenario where there is no dependence. Here we report the results for two scenarios as they represent the best- and worst-case computational scenarios for the MultiFIT procedure and defer the rest of the scenarios to the Supplementary Material. The first scenario we report involves data generated under the null hypothesis, with all margins drawn independently from a standard normal distribution. Under the second scenario, one dimension of *Y* is strongly correlated with a dimension of *X* under the linear scenario from Table S1 in the Supplementary Material with *l* = 3. While in practice nonlinear alternatives are the main motivation for the nonparametric tests being considered here, the linear scenario is essentially the worst-case scenario for the multi-scale Fisher’s independence test, without early stopping, in terms of computational time. The reason is that the stronger the dependency at coarser levels, the more tests will be performed under the multi-scale Fisher’s independence test, because more tests will pass the *p*-value threshold at coarser levels. As such, these two scenarios represent the two ends of the spectrum in the amount of computation incurred under the multi-scale Fisher’s independence test.

**Fig. 5.**
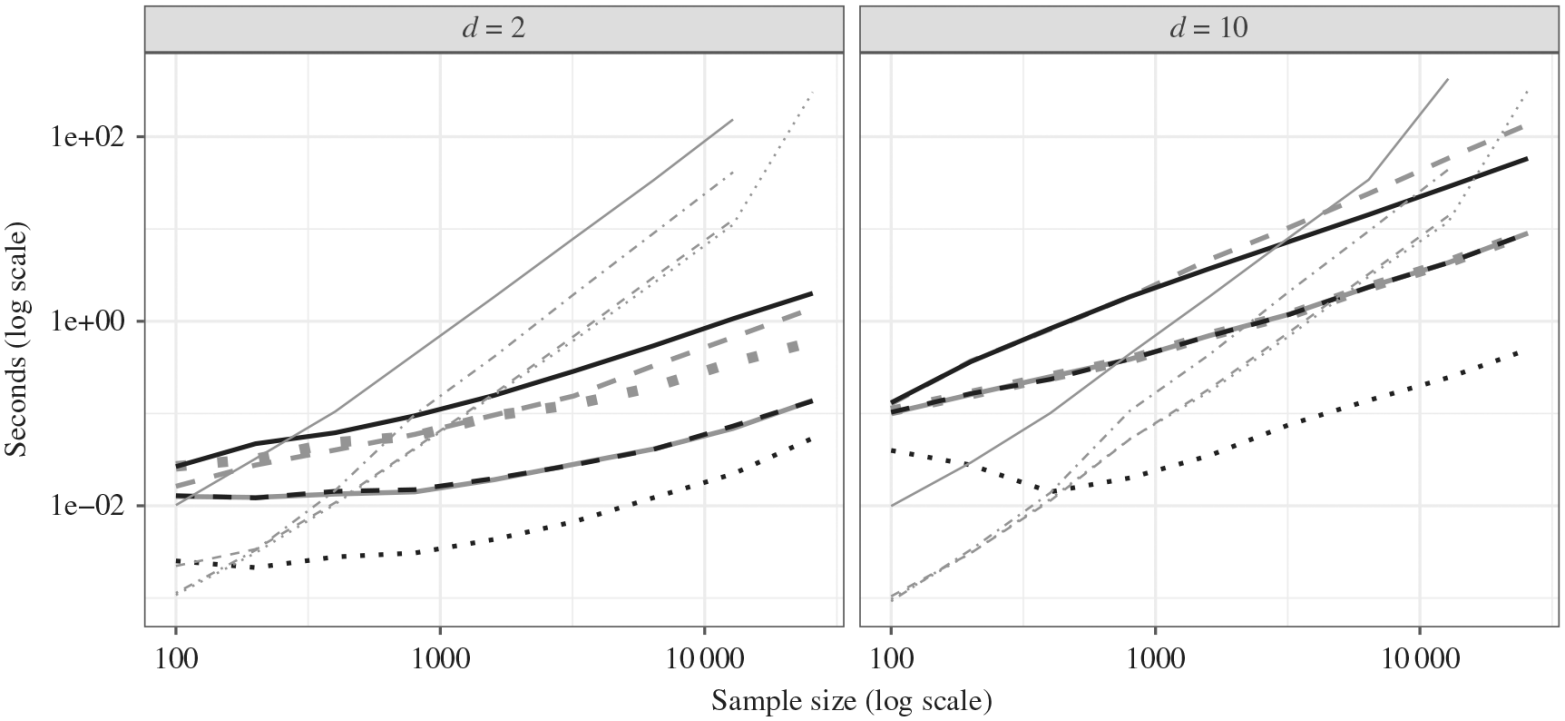
Computational scalability: a comparison of the Heller–Heller–Gorfine test (thin grey solid), distance covariance (thin grey dash–dot), Brownian distance covariance (thin grey dashed), Brownian distance covariance with the gamma approximation (thin grey dotted) and the multi-scale Fisher’s independence test. Three variants of the MultiFIT procedure were tested on two scenarios: the null scenario was tested with the full method (thick grey solid), the approximate method that keeps up to 100 most significant *p*-values at each resolution (thick grey dotted) and the full algorithm with early stopping (thick black dashed); a linear scenario was tested with the full method (thick grey dashed), the approximate method that keeps up to 100 most significant *p*-values at each resolution (thick black solid) and the full algorithm with early stopping (thick black dotted). In all cases *D*_*x*_ = *D*_*y*_ = *d*. The MultiFIT procedure was run with R* = 1 and p* = {*D*_*x*_*D*_*y*_ log_2_(*n*)}^−1^. The multi-scale Fisher’s independence test and the Brownian distance covariance method with gamma approximation do not require permutations. The other methods require permutations for level control and the reported time is for a single permutation.

Figure 5 plots the computational time versus the sample size on a log-log scale at different dimensionalities, 2 and 10. All methods were run on the same desktop computer with a single Intel^®^ Core^™^ i7-3770 CPU core at 3.40 GHz, and the three competitors to MultiFIT were evaluated up to the maximum sample size allowed by the available 16G RAM. We present the average duration of 10 executions of each method under different dimensions, *d* = 2 and *d* = 10. The results for the competitors are for only a single permutation, while at least hundreds of resampling repetitions are required in order to perform inference.

Overall, the computational advantage of the multi-scale Fisher’s independence test is substantial, as it scales approximately *O*(*n* log *n*) in sample size, while the Heller–Heller–Gorfine test, distance covariance and Brownian distance covariance without the gamma approximation scale approximately *O*(*n*^2^). The gamma approximation method of Brownian distance covariance makes the method faster in the presence of a strong signal, but it still cannot handle the larger sample sizes due to its memory requirement.

The multi-scale Fisher’s independence test with early stopping achieved the best computational efficiency at moderate to large sample sizes uniformly across nonnull scenarios. As expected, early stopping does not reduce computation under the null. The approximate multi-scale Fisher’s independence test with a maximum number of cuboids per resolution on the other hand bounds the complexity by *O*(*n* log *n*).

We do acknowledge that the three competitors scale linearly in dimensionality while the multi-scale Fisher’s independence test scales quadratically with the number of dimensions. As such, the multi-scale Fisher’s independence test is not suited for very high-dimensional problems. It is most suitable for problems up to tens of dimensions with large sample size.

### 3.2. Power comparison

We next examine the statistical power of the competing methods under several representative dependency scenarios. We consider two sets of simulation settings. In one set, the dependency exists only in a small number of margins, and thus is amenable to the multi-scale Fisher’s independence test’s search over pairs of axes-aligned boundaries. In the other set, the dependency is spread over a large number of dimensions and thus is particularly adversarial to the multi-scale Fisher’s independence test.

**Fig. 6.**
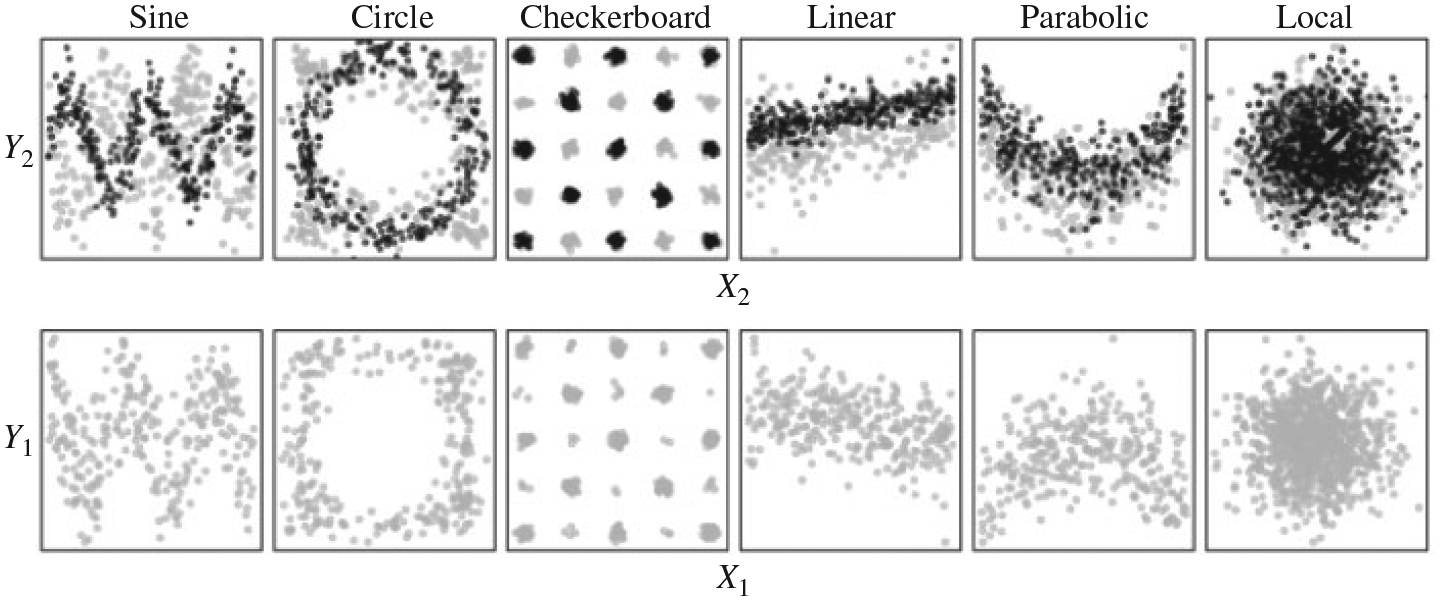
Visualization of the dependent margins of six scenarios with noise level 2. The marginal scenario (black dots) is only plotted in the top row as its *X*_1_-*Y*_1_ margins do not involve an interesting dependency, whereas the spread scenario (grey dots) is plotted in both. The dependency in the marginal scenario is more noticeable in the *X*_2_-*Y*_2_ margins than the spread scenario.

In the first set of simulations, we let *X*_1_ and *Y*_1_ be independently normally distributed, whereas *X*_2_ and *Y*_2_ are dependent according to several different scenarios, which are illustrated in Fig. 6 by black points in the upper row of plots. The multi-scale Fisher’s independence test has a natural advantage to detect such marginal dependencies as it focuses on the testing of pairs of margins.

In the second set of dependency scenarios, the true signal embodies dependencies of the *Y* margins on multiple *X* margins in terms of linear combinations or mixtures. This dissipates the strength of the dependency over many pairs of margins, and is thus highly unfavourable to the multi-scale Fisher’s independence test. This set of scenarios is illustrated in Fig. 6 by grey points in the two rows of plots and detailed in the Supplementary Material.

For all scenarios except the local scenarios, we set the level of resolutions up to which exhaustive testing is done, R* = 2, and for the local scenarios, where a signal is embedded in a small portion of the sample space, we set R* = 4 to ensure exhaustive coverage up to resolution 4. In the Supplementary Material we present a detailed sensitivity analysis on the effects of the tuning parameters p* and R* on the power of the test under the simulation settings.

We performed 500 simulations for each scenario at 20 different noise levels, and applied the four methods at the 5% level. We first applied a rank transform to each of the *D* margins for the simulated data, as this is the default under the MultiFIT procedure, and the competitors, the Heller–Heller–Gorfine test, distance covariance and Brownian distance covariance, also performed much better with the marginal rank transform.

**Fig. 7.**
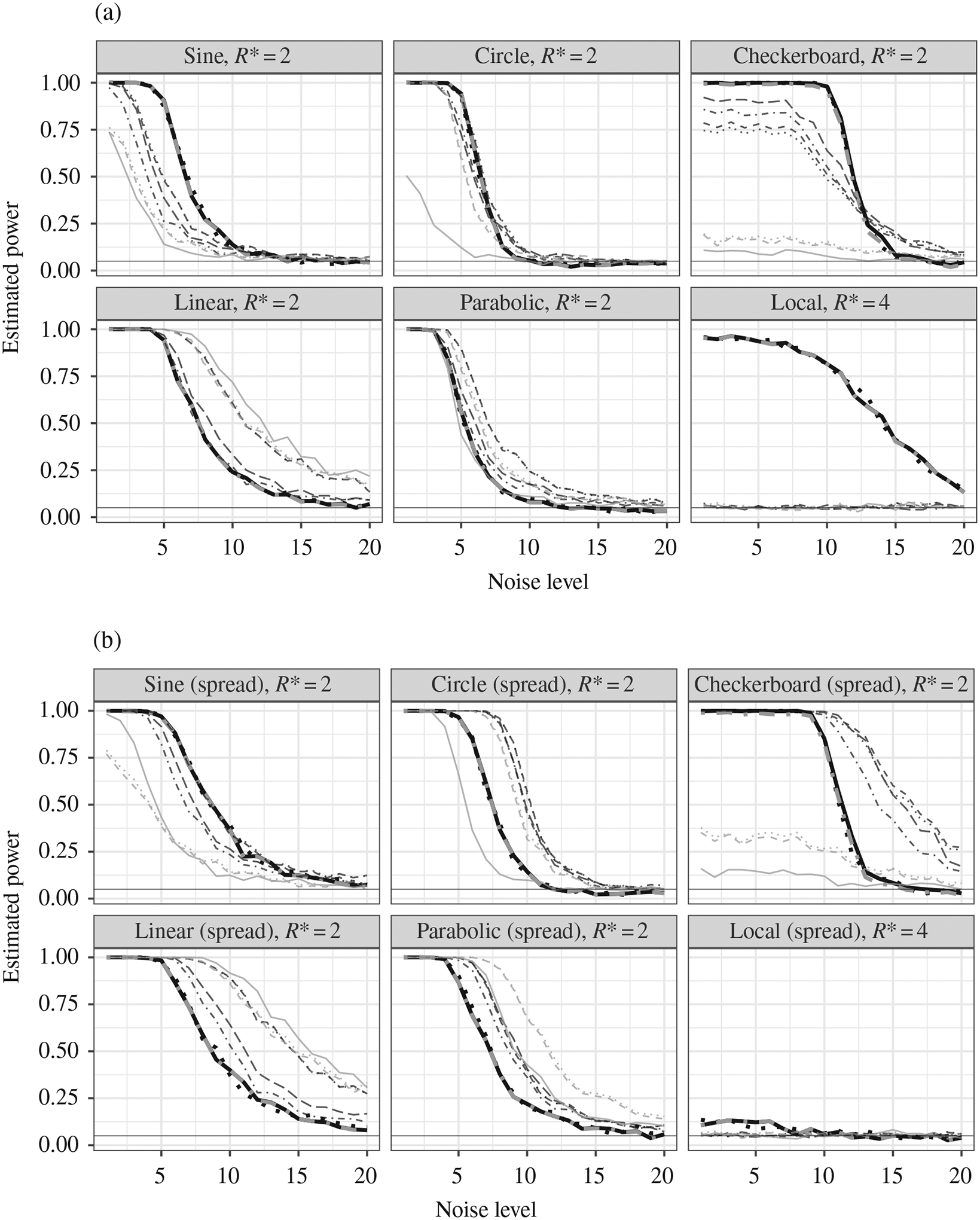
Power versus noise level for different methods. (a) Estimated power at 20 noise levels for the different methods under the six scenarios from Table S1 in the Supplementary Material. (b) Estimated power at 20 noise levels for the different methods under the six scenarios from Tables S2 and S3 in the Supplementary Material. In both panels we show three variants of the multi-scale Fisher’s independence test: the full method (thick black solid), the approximate method that keeps up to 100 most significant *p*-values at each resolution (thick black dotted) and the full algorithm with early stopping (thick grey dash-dot); four variants of the Heller–Heller–Gorfine test: the sum chi squared (thin black dashed), sum likelihood (thin black dotted), mean chi squared (thin black dash-dot) and mean likelihood (thin black long-dash); the distance covariance (thin grey solid line); the Brownian distance covariance (thin grey dotted) and the Brownian distance covariance with the gamma approximation (thin grey dashed).

Figure 7(a) reports the result for the first set of simulations. The MultiFIT procedure outperforms the Heller–Heller–Gorfine test, distance covariance and Brownian distance covariance for the sine, circle, checkerboard and local scenarios, the cases that are richer with local structures. For the more global dependency structures, namely linear and parabolic, the Heller–Heller–Gorfine test and Brownian distance covariance outperform the MultiFIT procedure, while distance covariance does so in the linear case. This is explained by the fact that the signal is observable almost entirely in the coarsest level, and as we go into higher resolutions we merely add insignificant tests that reduce the overall power. In the second set of simulations shown in Fig. 7(b), as expected, the MultiFIT procedure loses some power relative to the competitors. Nevertheless, its overall performance is still robust and it still outperforms all other methods in the sine and local spread scenarios.

The results are largely consistent with our intuition. Because of its divide-and-conquer nature, the multi-scale Fisher’s independence test is particularly good in identifying dependency structures that concentrate within a small number of cuboids, i.e., local features, while its power is weaker when the dependency structure is spread over a large number of cuboids, i.e., global structures.

Finally, we acknowledge that the performance of some of the competitors, such as the Brownian distance covariance, could be further improved with more expert selection of the tuning parameters. For example, the incorporation of a multi-scale bandwidth into the Brownian distance covariance, as suggested by Li &Yuan (2019), could further improve its performance.

### 3.3. Learning the nature of the dependency

So far, we have focused on applying the multi-scale Fisher’s independence test for testing the null hypothesis of independence. In practice, especially in multivariate settings, the practitioner is often interested in not just testing the existence of dependence, but in having an understanding of its nature. A by-product of the divide-and-conquer approach is the ability to shed light on the underlying dependency structure. In this section we provide two examples that illustrate the ability of the multi-scale Fisher’s independence test to learn the nature of the dependency. In the first example, we consider a dependency structure resulting from higher-order interactions. In the second example, the dependency consists of two sine waves in the (*X*_1_, *Y*_1_) margin with different frequencies, while a third margin, *X*_2_, determines the frequency. In both examples it is difficult to visualize the dependency in low-dimensional marginal visualizations. We show that after identifying the 2 × 2 tables that contained statistically significant evidence for dependency after multiple testing correction, by plotting the data points in those significant tables, one can learn and visualize the underlying dependency. In both examples, we use the holistic approach to multiple testing and adopt Holm’s correction on the *p*-values.

#### Example 1 (Rotated three-dimensional circle).

Let *X* and *Y* each be of three dimensions, and simulate a sample with 800 observations. We first generate a circle scenario so that *X*_1_, *Y*_1_, *X*_2_ and *Y*_2_ are all independent and identically distributed standard normals, whereas *X*_3_ = cos(*θ*) + *ϵ*, *Y*_3_ = sin(*θ*) + *ϵ*′ where *ϵ* and *ϵ*′ are independent and identically distributed *N*{0, (1/10)^2^} and *θ* ~ Un(−π, π). We then rotate the circle by *π*/4 degrees in the *X*_2_-*X*_3_-*Y*_3_ space by applying

$$\left[\begin{array}{ccc} \text{cos}(\pi /4) & -\text{sin}(\pi /4) & 0\\ \text{sin}(\pi /4) & \text{cos}(\pi /4) & 0\\ 0 & 0 & 1 \end{array}\right]\left[\begin{array}{ccc} | & | & |\\ X_{2} & X_{3} & Y_{3}\\ | & | & | \end{array}\right].$$

**Fig. 8.**
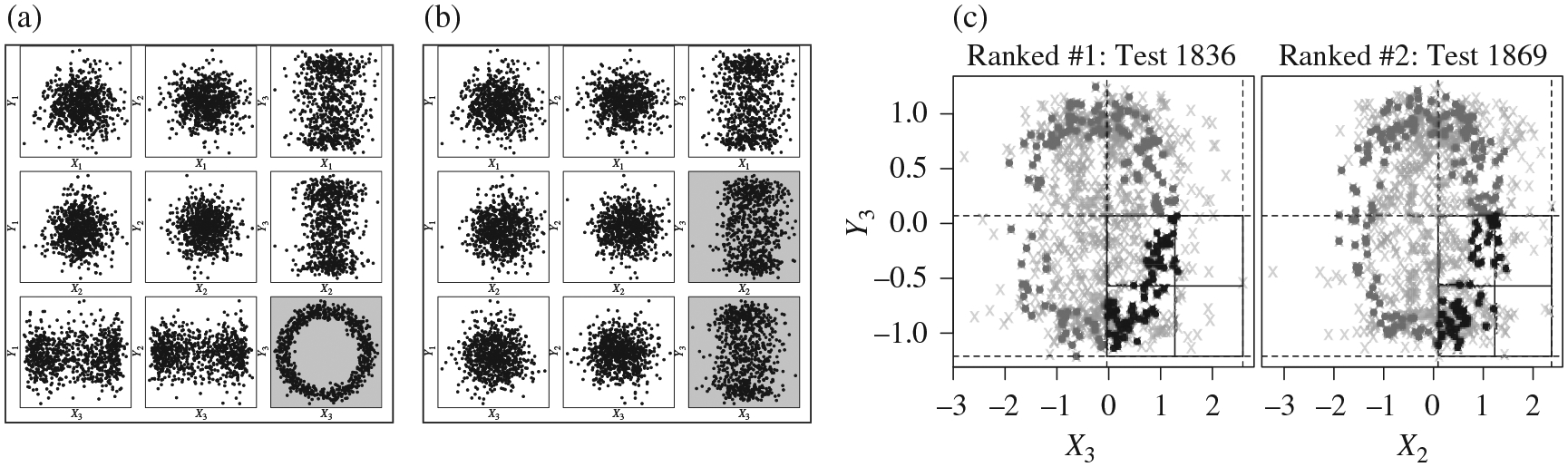
(a), (b) Marginal views of the data sample in § 3.3 (a) before and (b) after rotation. The dependency is easily visible in the marginal plots before rotation. Once rotated, the signal is spread among the margins and no longer visually obvious. (c) Scatter plots for the observations in the three 2 × 2 tables identified as most significant by the MultiFIT procedure for the rotated circle scenario. Significant tables are those with Holm’s adjusted *p*-values below 0.001. The dependency structure is again visible in the marginal views: black points are observations that are within the cuboid that is tested, dark-grey points are observations that are in a cuboid formed by expanding the tested cuboid so that the plotted margins are not subsetted and light grey crosses are all other data points. Note how the left plot captures the dependency in the *X*_3_-*Y*_3_ plane while the right plot captures the dependency in the *X*_2_-*Y*_3_ plane.

The rotated circle is no longer visible by examining the two-dimensional margins. See Fig. 8 for the marginal views of the sample before and after the rotation. Figure 8(c) plots the data points that lie in the 2 × 2 tables identified as statistically significant at the 0.001 level after multiple testing adjustment with the modified Holm’s procedure under the rotated setting. The underlying dependency pattern is clearly visible after selecting these tables. We found that in visualizing the identified tables, it is often useful to plot the data points that lie in the same slice of that table, but with the full ranges of the plotted margins, as the identified table often captures a portion of the interesting dependency. Figure 8(c) demonstrates this technique by plotting those additional observations in dark grey. For this reason, we have incorporated this plotting feature in our software.

#### Example 2 (Mixed sine signals).

Here we examine the ability of the multi-scale Fisher’s independence test to detect a dependency structure consisting of two sine waves in different frequencies. Let *X* = (*X*_1_, *X*_2_)^⊤^ be a two-dimensional random vector with independent margins *X*_1_ ~ *U*(0, 1) and *X*_2_ ~ Be(0.3, 0.3), and let

$$Y=\{\begin{array}{l} \text{sin}\left(10X_{1}\right)+\epsilon \text{ if }X_{2}>0.5,\\ \text{sin}\left(40X_{1}\right)+\epsilon \text{ if }X_{2}⩽0.5. \end{array}$$

**Fig. 9.**
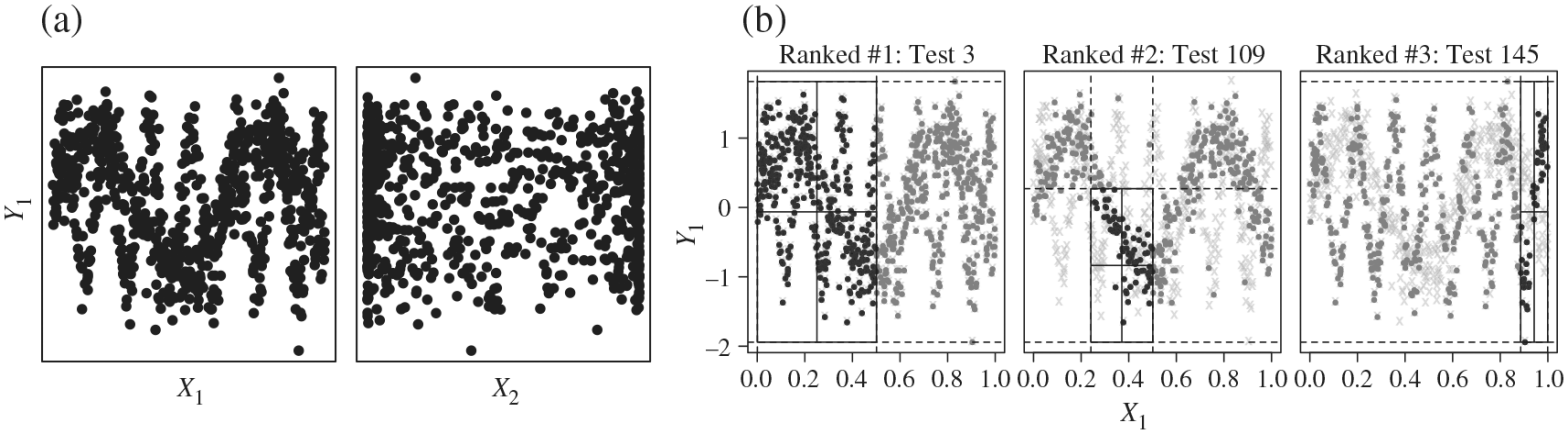
(a) The two pairs of margins of the sine mixture (black points). In the *X*_1_-*Y*_1_ plane we see the superimposed sine signals and in the *X*_2_-*Y*_1_ plane the margins that determine the mixture. (b) Scatter plots for the observations in the three 2 × 2 tables identified as most significant by the MultiFIT procedure for the sine mixture scenario. The black points are observations that are within the cuboid that is tested, dark grey points are observations that are in a cuboid formed by expanding the tested cuboid so that the plotted margins are not subsetted and light grey crosses are all other data points.

Figure 9(a) shows a simulated dataset of size 800. In the (*X*_1_, *Y*_1_) margin in the left panel we can see the superimposed sine waves. Figure 9(b) shows three significant tables identified by the MultiFIT procedure using the same colour coding technique from the previous example in which we can clearly discern between the different frequency waves.

## 4. Application to a flow cytometry dataset

Flow cytometry is the standard biological assay used to measure single cell features known as markers, and is commonly used to quantify the relative frequencies of cell subsets in blood or disaggregated tissue. These features may be general physical, chemical or biological properties of a cell. Such data involve complex distributional features and are of massive sizes with typical sample sizes in the range of hundreds of thousands, which presents computational challenges to nonparametric data analytical tools.

For the evaluation, we used flow cytometry samples generated by an antibody panel designed to identify activated T cell subsets. We show the results of the dependency analysis on a single illustrative sample with 353 586 cells. For the analysis, we separated the markers into a vector of four basic markers, CD3, CD4, dump and CD8, and a vector of four functional markers, IFN, TNF, IL-2 and CD107. The basic markers are used in practice to first identify viable T cells by exclusion using the dump and CD3 markers, and then to further partition T cells into CD4-positive helper and CD8-positive cytotoxic subsets. The functional markers are used to identify the activation status of these T cell subsets and their functional effector capabilities. Here, IL-2 is a T cell growth factor, IFN and TNF are inflammatory cytokines, and CD107 is a component of the mechanism used by T cells to directly kill infected and cancer cells.

We applied the MultiFIT procedure with Holm’s multiple testing adjustment to the data to identify dependency between the basic and functional markers. Our aim here is to demonstrate the ability of the multi-scale Fisher’s independence test to handle such large data and to shed light on the underlying dependency, and so we ran the test exhaustively up to the maximal resolution of 4, testing 102 416 2 × 2 tables. The execution time of the algorithm in this setting is approximately five minutes on a laptop computer utilizing four 3.00 GHz Intel^®^ Xeon^®^ E3-1505M v6 CPU cores.

**Fig. 10.**
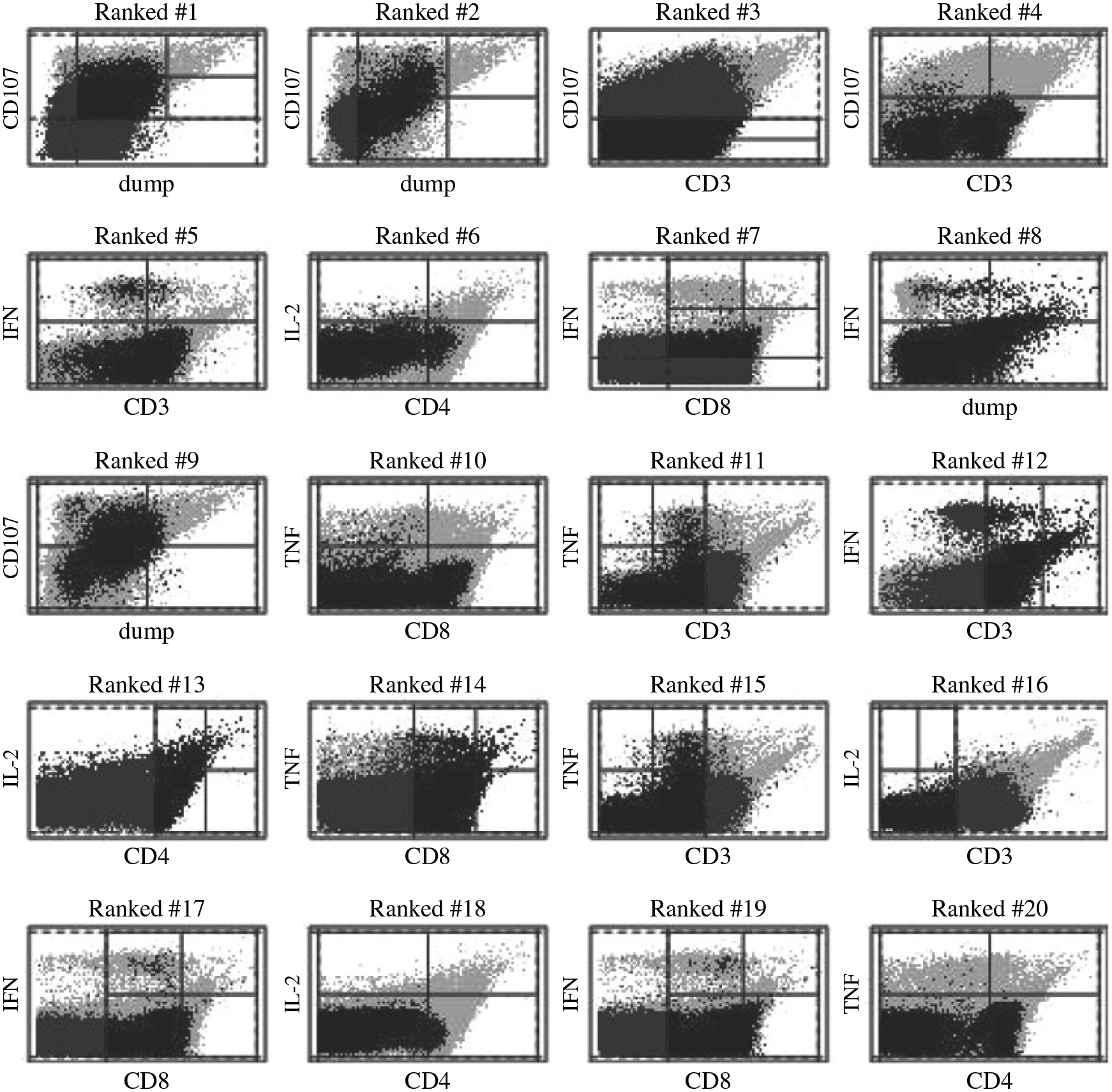
Scatter plots of the observations identified by the 20 2 × 2 tables with the most significant *p*-values for the flow cytometry dataset. Black indicates observations in the tested cuboid. Dark grey indicates observations in the same slice of the sample space, determined by the four markers other than the two margins plotted. Light grey indicates the rest of the observations.

As the sample size is very large and the data clearly have strong marginal dependencies, the MultiFIT procedure identified hundreds of significant tests after multiple testing adjustment. Interested readers can run our code for this example in the Supplementary Material to visualize the identified dependence structures. The Heller–Heller–Gorfine test, distance covariance and Brownian distance covariance were not able to handle this amount of data and all ended in overflow errors. Figure 10 presents the visualization of the observations in the 20 2 × 2 table with the most significant *p*-values using the strategy described in § 3.3.

## Supplementary Material

Supplementary Material
