# Supplementary material for: Multi-scale Fisher’s independence test for multivariate dependence
Source: Biometrika. Author manuscript; Available in PMC 2022 Nov 14. (PMC9648765; doi:10.1093/biomet/asac013)
Supplement: Supplementary Material [file NIHMS1821790-supplement-Supplementary_Material.pdf]

Biometrika (2021), **103**, 1, *pp.* 1–24
Printed in Great Britain Advance Access publication on 31 July 2018

Supplementary material for "Multiscale Fisher's Independence Test for Multivariate Dependence"

BY S. GORSKY
Department of Mathematics and Statistics, University of Massachusetts Amherst, Amherst, Massachusetts 01003, U.S.A.
sgorsky@umass.edu

AND L. MA
Department of Statistical Science, Duke University, Durham, North Carolina 27708, U.S.A.
li.ma@duke.edu

1. TECHNICAL PROOFS

In this section, we establish proofs for Theorem 2.1, Theorem 2.2, and Corollary 2.1 in the main paper. We will introduce a number of definitions and lemmas along the way that will be used to complete the proofs.

DEFINITION S1 (LEVEL-k CANONICAL MARGINAL PARTITION). *$\mathcal{P}^k$ is a level-k canonical marginal partition if*

$$\mathcal{P}^k = \left\{ \left[\frac{l-1}{2^k}, \frac{l}{2^k} \right) \right\}_{l \in \{1,\dots,2^k\}}.$$

To simplify the notations in the proofs, we let $Z = (X, Y)$. More specifically, we set $Z_1 = X_1, ..., Z_{D_x} = X_{D_x}$ and $Z_{D_x+1} = Y_1, ..., Z_D = Y_{D_y}$. Thus, Z is a random vector that distributed according to the distribution F, the joint sampling distribution of (X, Y).

For $k = (k_1, ..., k_D) \in \mathbb{N}_0^D$, we refer to the partition $\mathcal{P}^{k_1} \times \cdots \times \mathcal{P}^{k_D}$ as the k-*stratum* of Ω. Denote now any k-*stratum* as $\mathcal{A}^k$. As described in Section 2, we form D-dimensional cuboids by taking the Cartesian product of one interval from each of the D canonical marginal partitions of Ω. Given then $k = (k_1, ..., k_D) \in \mathbb{N}_0^D$ a specific cuboid is determined by some $l = (l_1, ..., l_D)$ with each $1 \le l_d \le 2^{k_d}$ such that $A = \bigtimes_{d \in \{1,..,D\}} \left[(l_d - 1)/2^{k_d}, l_d/2^{k_d}\right)$. Figure S1 illustrates these definitions in a three dimensional space.

We next define a discretized form of independence which we will show in Lemma S1 fully characterizes the multivariate independence:

DEFINITION S2 (k-INDEPENDENCE). *For any $A \in \mathcal{A}^k$, we can write $A = A_x \times A_y$ where*

$$A_x = \bigtimes_{d=1}^{D_x} \left[\frac{l_d - 1}{2^{k_d}}, \frac{l_d}{2^{k_d}} \right) \text{ and } A_y = \bigtimes_{d=D_x+1}^{D} \left[\frac{l_d - 1}{2^{k_d}}, \frac{l_d}{2^{k_d}} \right).$$

Figure S2 illustrates the above notations in three dimensional space.

We say that X and Y are k-independent and write it as $X \perp\!\!\!\perp_k Y$ if for any $A \in \mathcal{A}^k$,

$$\text{pr}\left(X \in A_x, Y \in A_y\right) = \text{pr}\left(X \in A_x\right) \cdot \text{pr}\left(Y \in A_y\right).$$

© 2021 Biometrika Trust

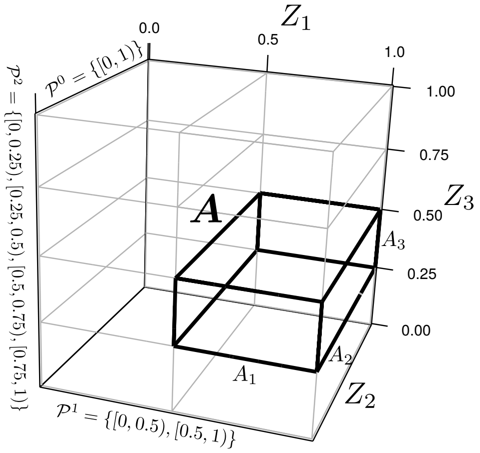

Fig. S1. Cuboid and stratum.
A 3D view of a k-stratum and the cuboid A where $D = 3$ and $k = (1, 0, 2)$. The thin grey lines delineate all $(1, 0, 2)$-cuboids, that is, the stratum $\mathcal{A}^{(1,0,2)} = \mathcal{P}^1 \times \mathcal{P}^0 \times \mathcal{P}^2$. The thick black lines delineate the cuboid A for which $l = (2, 1, 2)$, i.e., $A = A_1 \times A_2 \times A_3$ where $A_1 = \big[(2-1)/2^1, 2/2^1\big) = [0.5, 1) \in \mathcal{P}^1$, $A_2 = \big[(1-1)/2^0, 1/2^0\big) = [0, 1) \in \mathcal{P}^0$, and $A_3 = \big[(2-1)/2^2, 2/2^2\big) = [0.25, 0.5) \in \mathcal{P}^2$. The resolution of this stratum is $3 = 1 + 0 + 2$.

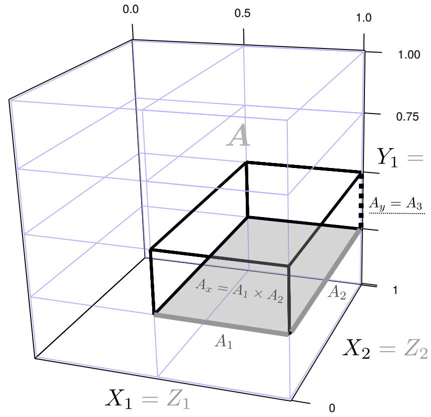

Fig. S2. A_x and A_y.
A 3D view of a k-stratum and the cuboid A where $D = 3$, $k = (1, 0, 2)$ and $l = (2, 1, 2)$, the same as in Fig. S1. Here $D_x = 2$ with $X_1 = Z_1$ and $X_2 = Z_2$; $D_y = 1$ with $Y_1 = Z_3$. The cuboid A is represented now as $A = A_x \times A_y$ where $A_x = A_1 \times A_2 = \big[(2-1)/2^1, 2/2^1\big) \times \big[(1-1)/2^0, 1/2^0\big) = [0.5, 1) \times [0, 1)$, and $A_y = A_3 = \big[(2-1)/2^2, 2/2^2\big) = [0.25, 0.5)$.

DEFINITION S3 ((i, j)-BLOCKS OF A CUBOID). *For every cuboid $A \in \mathcal{A}^k$, $i \in \{1, .., D_x\}$ and $j \in \{1, .., D_y\}$, one can partition A into four blocks by dividing A in the (i, j)th face, i.e.*

the side of A spanned by the ith and jth dimensions, while keeping the other dimensions intact.

$$A = A_{ij}^{00} \cup A_{ij}^{01} \cup A_{ij}^{10} \cup A_{ij}^{11},$$

where for $a, b \in \{0, 1\}$,

$$A_{ij}^{ab} = \bigtimes_{d=1}^{D} \begin{cases} \left[\frac{2l_d-2+a}{2^{k_d+1}}, \frac{2l_d-1+a}{2^{k_d+1}}\right) & \text{if } d = i \\ \left[\frac{2l_d-2+b}{2^{k_d+1}}, \frac{2l_d-1+b}{2^{k_d+1}}\right) & \text{if } d = D_x + j \\ \left[\frac{l_d-1}{2^{k_d}}, \frac{l_d}{2^{k_d}}\right) & \text{if } d \in \{1, .., D\} \setminus \{i, D_x + j\} \end{cases}.$$

Figure S3 illustrates Definition S3 in three dimensions.

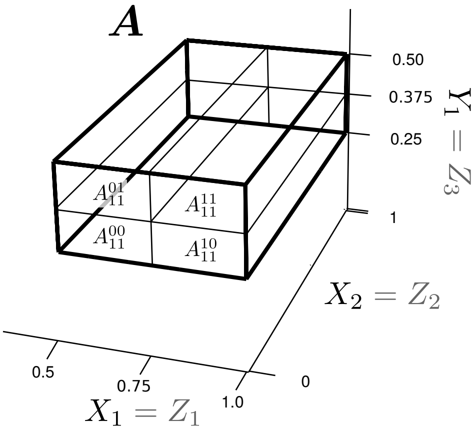

Fig. S3. ($i = 1, j = 1$) blocks of A.
A 3D view of the k-cuboid A where $D = 3$, $k = (1, 0, 2)$ and $l = (2, 1, 2)$, the same as in Fig. S1 and Fig. S2. The $i = 1$ dimension of X corresponds to dimension 1 of Z the $j = 1$ dimension of Y corresponds to dimension 3 of Z. The blocks are:

$A_{11}^{00} = \big[(2 \cdot 2 - 2 + 0)/2^{1+1}, (2 \cdot 2 - 1 + 0)/2^{1+1}\big) \times \big[(1-1)/2^0, 1/2^0\big) \times \big[(2 \cdot 2 - 2 + 0)/2^{2+1}, (2 \cdot 2 - 1 + 0)/2^{2+1}\big) = [0.5, 0.75) \times [0, 1) \times [0.25, 0.375),$

$A_{11}^{01} = \big[(2 \cdot 2 - 2 + 0)/2^{1+1}, (2 \cdot 2 - 1 + 0)/2^{1+1}\big) \times \big[(1-1)/2^0, 1/2^0\big) \times \big[(2 \cdot 2 - 2 + 1)/2^{2+1}, (2 \cdot 2 - 1 + 1)/2^{2+1}\big) = [0.5, 0.75) \times [0, 1) \times [0.375, 0.5),$

$A_{11}^{10} = \big[(2 \cdot 2 - 2 + 1)/2^{1+1}, (2 \cdot 2 - 1 + 1)/2^{1+1}\big) \times \big[(1-1)/2^0, 1/2^0\big) \times \big[(2 \cdot 2 - 2 + 0)/2^{2+1}, (2 \cdot 2 - 1 + 0)/2^{2+1}\big) = [0.75, 1) \times [0, 1) \times [0.25, 0.375),$

$A_{11}^{11} = \big[(2 \cdot 2 - 2 + 1)/2^{1+1}, (2 \cdot 2 - 1 + 1)/2^{1+1}\big) \times \big[(1-1)/2^0, 1/2^0\big) \times \big[(2 \cdot 2 - 2 + 1)/2^{2+1}, (2 \cdot 2 - 1 + 1)/2^{2+1}\big) = [0.75, 1) \times [0, 1) \times [0.375, 0.5).$

Lemma S1 establishes an equivalence between multivariate independence and a cascade of discretized multivariate independence relations:

LEMMA S1.

$$X \perp\!\!\!\perp Y \quad \Leftrightarrow \quad X \perp\!\!\!\perp_k Y \quad \textit{for all } k \in \mathbb{N}_0^D.$$

Proof. $\Rightarrow$: Immediate.
$\Leftarrow$:
Let

$$\mathcal{P}_x = \bigcup_{k_{\{1,\dots D_x\}} \in \mathbb{N}_0^{D_x}} \mathcal{P}^1 \times \dots \mathcal{P}^{D_x}.$$

Therefore $\sigma(\mathcal{P}_x) = \mathcal{B}([0,1]^{D_x})$.

For $A_x \in \mathcal{B}([0,1]^{D_x})$ let $[X \in A_x] = \{\omega : X(\omega) \in A_x\}$, then $\sigma(X) = \{[X \in A_x], A_x \in \mathcal{B}([0,1]^{D_x})\}$.

Let

$$\mathcal{Q}_x = \bigcup_{k_{\{1,\dots D_x\}} \in \{-1,0,1,2,\dots\}^{D_x}} \mathcal{Q}^{k_1} \times \dots \times \mathcal{Q}^{k_{D_x}}$$

so that $\mathcal{Q}^{-1} := \emptyset$ and for all $k \geq 0$, $\mathcal{Q}^k = \mathcal{P}^k$.

Let $\mathcal{C}_X = \{[X \in B_x], B_x \in \mathcal{Q}_x\}$.

Hence $\sigma(\mathcal{C}_X) = \sigma\{X^{-1}(B_x), B_x \in \mathcal{Q}_x\} = \sigma\{X^{-1}(\mathcal{Q}_x)\} = X^{-1}\{\sigma(\mathcal{Q}_x)\} = X^{-1}\{\sigma(\mathcal{P}_x)\} = \sigma(X)$.

$\mathcal{C}_X$ is a π-system:

$$E, E' \in \mathcal{C}_X \quad \Rightarrow \quad E = \left\{X \in \left(\emptyset \text{ or } \left[\frac{l_1 - 1}{2^{k_1}}, \frac{l_1}{2^{k_1}}\right)\right) \times \dots \times \left(\emptyset \text{ or } \left[\frac{l_{D_x} - 1}{2^{k_{D_x}}}, \frac{l_{D_x}}{2^{k_{D_x}}}\right)\right)\right\}$$

and

$$E' = \left\{X \in \left(\emptyset \text{ or } \left[\frac{l'_1 - 1}{2^{k'_1}}, \frac{l'_1}{2^{k'_1}}\right)\right) \times \dots \times \left(\emptyset \text{ or } \left[\frac{l'_{D_x} - 1}{2^{k'_{D_x}}}, \frac{l'_{D_x}}{2^{k'_{D_x}}}\right)\right)\right\}$$

for some l, l', k, k'. Therefore:

$$\begin{aligned} E \cap E' = \Bigg[X \in &\left\{\left(\emptyset \text{ or } \left[\frac{l_1 - 1}{2^{k_1}}, \frac{l_1}{2^{k_1}}\right)\right) \times \dots \times \left(\emptyset \text{ or } \left[\frac{l_{D_x} - 1}{2^{k_{D_x}}}, \frac{l_{D_x}}{2^{k_{D_x}}}\right)\right)\right\} \bigcap \\ &\left\{\left(\emptyset \text{ or } \left[\frac{l'_1 - 1}{2^{k'_1}}, \frac{l'_1}{2^{k'_1}}\right)\right) \times \dots \times \left(\emptyset \text{ or } \left[\frac{l'_{D_x} - 1}{2^{k'_{D_x}}}, \frac{l'_{D_x}}{2^{k'_{D_x}}}\right)\right)\right\}\Bigg] \\ = \Bigg\{X \in &\left(\emptyset \text{ or } \left[\frac{l_1 - 1}{2^{k_1}}, \frac{l_1}{2^{k_1}}\right) \cap \left[\frac{l'_1 - 1}{2^{k'_1}}, \frac{l'_1}{2^{k'_1}}\right)\right) \times \dots \\ &\times \left(\left[\frac{l_{D_x} - 1}{2^{k_{D_x}}}, \frac{l_{D_x}}{2^{k_{D_x}}}\right) \cap \left[\frac{l'_{D_x} - 1}{2^{k'_{D_x}}}, \frac{l'_{D_x}}{2^{k'_{D_x}}}\right)\right)\Bigg\} \in \mathcal{C}_X \end{aligned}$$

Similarly, define $\sigma(Y)$ and $\mathcal{C}_Y$, another π-system, independent of $\mathcal{C}_X$ and $\sigma(Y) = \sigma(\mathcal{C}_Y)$. By the basic criterion, then, $\sigma(X) \perp\!\!\!\perp \sigma(Y)$. □

The following lemma shows that if X and Y are k-independent, they are also independent on all coarser strata.

LEMMA S2. *If* $X \perp\!\!\!\perp_k Y$ *then* $X \perp\!\!\!\perp_{k'} Y$ *for all* $k' \leq k$.

Proof. Let F_X be the probability distribution of X and F_Y be the probability distribution of Y. Then $X \perp\!\!\!\perp_k Y \quad \Leftrightarrow \quad F(A) = F_X(A_x)F_Y(A_y)$ for all $A \in \mathcal{A}^k$.

It is enough to show that $X \perp\!\!\!\perp_k Y \;\Rightarrow\; X \perp\!\!\!\perp_{k'} Y$ for any k' such that (i) $k'_i = k_i - 1$ for some $i \in \{1, ..., D_x\}$ and (ii) $k'_d = k_d$ for all $d \in \{1, .., D\} \setminus \{i\}$.

Hence, assuming $X \perp\!\!\!\perp_k Y$ we get for $A \in \mathcal{A}^{k'}$ that:

$$
\begin{aligned}
F(A) &= F\left(A_1 \times ... \times A_{i-1} \times A_i^0 \times A_{i+1} \times ... \times A_{D_x} \times A_y\right) \\
&\quad + F\left(A_1 \times ... \times A_{i-1} \times A_i^1 \times A_{i+1} \times ... \times A_{D_x} \times A_y\right) \\
&= F_X\left(A_1 \times ... \times A_{i-1} \times A_i^0 \times A_{i+1} \times ... \times A_{D_x}\right) F_Y(A_y) \\
&\quad + F_X\left(A_1 \times ... \times A_{i-1} \times A_i^1 \times A_{i+1} \times ... \times A_{D_x}\right) F_Y(A_y) \\
&= F_X(A_x) F_Y(A_y)
\end{aligned}
$$

As required. □

In Lemma S6 we will show how to characterize the multivariate k-independence with a collection of univariate (i, j) odds-ratios. However, before we state and prove Lemma S6 we develop additional notations and provide discretized versions of some basic results in probability.

Denote:
$d \subset \{1, .., D\}$, $i = d \cap \{1, .., D_x\}$ and $j = \{j^* : j^* + D_x \in d \cap \{D_x + 1, ..., D\}\}$
And

$$
\begin{aligned}
&X_i = \{X_{i^*} : i^* \in i\}, && X_{(i)} = \{X_{i'} : i' \in \{1, .., D_x\} \setminus i\} \\
&Y_j = \{Y_{j^*} : j^* \in j\}, && Y_{(j)} = \{Y_{j'} : j' \in \{1, .., D_y\} \setminus j\} \\
&Z_d = X_i \times Y_j = \{Z_{d'} : d' \in d\}, && Z_{(d)} = X_{(i)} \times Y_{(j)} = \{Z_{d'} : d' \in \{1, .., D\} \setminus d\}
\end{aligned}
$$

$$
\begin{aligned}
&A_{x,d} = \bigtimes_{d' \in d \cap \{1, .., D_x\}} A_{d'}, && A_{x,(d)} = \bigtimes_{d' \in \{1, .., D_x\} \setminus d} A_{d'} \\
&A_{y,d} = \bigtimes_{d' \in d \cap \{D_x+1, .., D\}} A_{d'}, && A_{y,(d)} = \bigtimes_{d' \in \{D_x+1, .., D\} \setminus d} A_{d'} \\
&A_d = A_{x,d} \times A_{y,d} = \bigtimes_{d' \in d} A_{d'}, && A_{(d)} = A_{x,(d)} \times A_{y,(d)} = \bigtimes_{d' \in \{1, .., D\} \setminus d} A_{d'}
\end{aligned}
$$

$$
k_d = \{k_{d'} : d' \in d\}, \qquad k_{(d)} = \{k_{d'} : d' \in \{1, .., D\} \setminus d\}
$$

DEFINITION S4 (CONDITIONAL k-INDEPENDENCE). *We say that X_i and Y_j are k-independent conditional on $Z_{(d)}$ and write it as $X_i \perp\!\!\!\perp_k Y_j \mid Z_{(d)}$ if for any $A \in \mathcal{A}^k$:*

$$
\mathrm{pr}(X_i \in A_{x,d}, Y_j \in A_{y,d} \mid Z_{(d)} \in A_{(d)}) = \mathrm{pr}(X_i \in A_{x,d} \mid Z_{(d)} \in A_{(d)}) \cdot \mathrm{pr}(Y_j \in A_{y,d} \mid Z_{(d)} \in A_{(d)})
$$

Or equivalently:

$$
\mathrm{pr}(X_i \in A_{x,d} \mid Y_j \in A_{y,d}, Z_{(d)} \in A_{(d)}) = \mathrm{pr}(X_i \in A_{x,d} \mid Z_{(d)} \in A_{(d)})
$$

When $k_{d'} = 0$ for some $d' \in \{1, .., D\} \setminus d$, $\Omega_{Z_{d'}} = [0, 1]$ for those indices and hence our notation may be compacted. For example, if $k_{d'} = 0$ for all $d' \in \{1, .., D\} \setminus d$:

$$
X_i \perp\!\!\!\perp_k Y_j | Z_{(d)} \;\Leftrightarrow\; X_i \perp\!\!\!\perp_{k_d} Y_j
$$

We next provide discretized versions of some basic results in probability:

LEMMA S3 (CONTRACTION). *For $d \subset \{1,..,D\}$ such that $\{1,..,D_x\} \subset d$, i.e. $i = \{1,..,D_x\}$, $X_i = X$ and $Y_{(j)} = Z_{(d)}$:*

$$\begin{cases} X \perp\!\!\!\perp_k Y_j \mid Z_{(d)} \\ X \perp\!\!\!\perp_{k_{(d)}} Y_{(j)} \end{cases} \Rightarrow X \perp\!\!\!\perp_k Y$$

Proof. Immediate from definition.

LEMMA S4 (DECOMPOSITION). *For $d \subset \{1,..,D\}$ such that $\{1,..,D_x\} \subset d$, i.e. $i = \{1,..,D_x\}$ and $X_i = X$:*

$$X \perp\!\!\!\perp_k Y \Rightarrow \begin{cases} X \perp\!\!\!\perp_{k_d} Y_j \\ X \perp\!\!\!\perp_{k_{(d)}} Y_{(j)} \end{cases}$$

Proof. Immediate from definition.

LEMMA S5 (WEAK UNION). *For $d \subset \{1,..,D\}$ such that $\{1,..,D_x\} \subset d$, i.e. $i = \{1,..,D_x\}$, $X_i = X$, $Y_j = Z_d$ and $Y_{(j)} = Z_{(d)}$:*

$$X \perp\!\!\!\perp_k Y \Rightarrow \begin{cases} X \perp\!\!\!\perp_k Y_j \mid Z_{(d)} \\ X \perp\!\!\!\perp_k Y_{(j)} \mid Z_d \end{cases}$$

Proof. Immediate from definition.

DEFINITION S5. *For $A_d \in \mathcal{P}^{k_d-1}$ such that $A_d = \left[(l_d-1)/2^{k_d-1}, l_d/2^{k_d-1}\right)$, define*

$$A_d^0 = \left[\frac{2l_d-2}{2^{k_d}}, \frac{2l_d-1}{2^{k_d+1}}\right) \in \mathcal{P}^{k_d}$$

and

$$A_d^1 = \left[\frac{2l_d-1}{2^{k_d+1}}, \frac{2l_d}{2^{k_d+1}}\right) \in \mathcal{P}^{k_d}$$

.

DEFINITION S6. *Given $k \in \mathbb{N}_0^D$ and $i \in \{1,..,D_x\}$ $j \in \{1,..,D_y\}$, let*

$$k[i,j] = \left\{k' \in \mathbb{N}_0^D : k'_i < k_i, k'_{D_x+j} < k_{D_x+j} \text{ and } k'_d \le k_d \text{ for all } d \in \{1,..,D\} \setminus \{i, D_x+j\}\right\}$$

and

$$\mathcal{A}^{k[i,j]} = \bigcup_{k' \in k[i,j]} \mathcal{A}^{k'}.$$

DEFINITION S7. *Given $k \in \mathbb{N}_0^D$ and $i \in \{1,..,D_x\}$, $j \in \{1,..,D_y\}$ let*

$$\begin{aligned}[k_i,k_j](k_{<i,<j}) = \{k' \in \mathbb{N}_0^D : \quad & k'_i < k_i, k'_{D_x+j} < k_{D_x+j} \text{ and} \\ & k'_d = k_d \text{ for all } d \in \{1,...,i-1\} \cup \{D_x+1,...,D_x+j-1\} \\ & \text{and} \\ & k'_d = 0 \text{ for all } d \in \{i+1,...,D_x\} \cup \{D_x+j+1,...,D\}\}\end{aligned}$$

Accordingly, let

$$\mathcal{A}^{[k_i,k_j](k_{<i,<j})} = \bigcup_{k' \in [k_i,k_j](k_{<i,<j})} \mathcal{A}^{k'},$$

which denotes the totality of all cuboids in strata that are coarser than the stratum $\mathcal{A}^k$ *along the margins i and j such that margins* $\{i+1, ..., D_x\}$ *and* $\{D_x + j + 1, ..., D\}$ *are allowed any value in* $[0, 1]$.

Lemma S6 ties the k-independence of the random vectors X and Y with the (i, j) odds-ratios:

LEMMA S6. *For any* $k > 0_D$*, where* 0_D *is a vector of 0s of length D*

$$X \perp\!\!\!\perp_k Y \quad \Leftrightarrow \quad \theta_{ij}(A) = 1 \quad \text{for all } i \in \{1, \dots, D_x\},\ \ j \in \{1, \dots, D_y\},\ \ A \in \mathcal{A}^{k'}$$
$$\text{for all } k' \in \mathbb{N}_0^D \text{ such that } k' \leq k \text{ with } k'_i < k_i \text{ and } k'_{D_x+j} < k_{D_x+j}.$$

Proof. $\Rightarrow$:
Assume $X \perp\!\!\!\perp_k Y$ for some $k \in \mathbb{N}_0^D$.
Let $i \in \{1, .., D_x\}$ and $j \in \{1, .., D_y\}$.
By applying the weak union lemma twice we get that $X_i \perp\!\!\!\perp_{(k_i, k_{D_x+j})} Y_j \mid Z_{(\{i, D_x+j\})}$.
For k' such that $k'_i = k_i - 1$, $k'_{D_x+j} = k_{D_x+j} - 1$, $k'_d = k_d$ for all $d \in \{1, .., D\} \setminus \{i, D_x + j\}$ and $A \in \mathcal{A}^{k'}$ we get that $A_{ij}^{00}, A_{ij}^{11}, A_{ij}^{01}, A_{ij}^{10} \in \mathcal{A}^k$. So $\theta_{ij}(A) = 1$ by the definitions of conditional independence and the (i, j) odds-ratios.
By Lemma S2 we get that indeed $\theta_{ij}(A) = 1$ for $A \in \mathcal{A}^{[k_i,k_j]}$.
$\Leftarrow$:
First, it suffices to show that:

$$X \perp\!\!\!\perp_k Y \quad \Leftarrow \quad \theta_{ij}(A) = 1$$
$$\text{for all } i \in \{1, 2, \dots, D_x\},\ \ j \in \{D_x + 1, \dots, D\},\ \ A \in \mathcal{A}^{[k_i,k_j](k_{<i,<j})}$$

Since then we may rely on the opposite direction to get that $\theta_{ij}(A) = 0$ for all $A \in \mathcal{A}^{[k_i,k_j]}$.
Examine

$$X_i \perp\!\!\!\perp_{k_{\{1,\dots,i,D_x+1,\dots,D_x+j\}}} Y_j \mid X_{\{1,\dots,i-1\}}, Y_{\{1,\dots,j-1\}}$$

To see that the above is true, let $k' \in \mathbb{N}_0^D$ such that $k'_d = k_d$ for all $d \in \{1, ..., i\} \cup \{D_x + 1, ..., D_x + j\}$ and
$k'_d = 0$ for all $d \in \{i + 1, ..., D_x\} \cup \{D_x + j + 1, ..., D\}$.
For a given $A \in \mathcal{A}^{k'}$ let x, y be a pair of univariate random variables whose joint distribution is given by $G := F_{X_i, Y_j \mid X_{\{1,\dots,i-1\}} \in A_{x,\{1,\dots,i-1\}}, Y_{\{1,\dots,j-1\}} \in A_{y,\{D_x+1,\dots,D_x+j-1\}}}$. By assumption:

$$1 = \theta_{ij}(A) = \frac{F(A_{ij}^{00}) \cdot F(A_{ij}^{11})}{F(A_{ij}^{01}) \cdot F(A_{ij}^{10})}$$
$$= \frac{G(A_i^0 \times A_{D_x+j}^0) \cdot G(A_i^1 \times A_{D_x+j}^1)}{G(A_i^0 \times A_{D_x+j}^1) \cdot G(A_i^1 \times A_{D_x+j}^0)}$$

Since the above holds for every $A \in \mathcal{A}^{[k_i,k_j](k_{<i,<j})}$ we may apply Theorem 2 from Ma and Mao (2019) and conclude that $x \perp\!\!\!\perp_{(k_i, k_{D_x+j})} y$ which is equivalent to $X_i \perp\!\!\!\perp_{k_{\{1,\dots,i,D_x+1,\dots,D_x+j\}}} Y_j \mid X_{\{1,\dots,i-1\}}, Y_{\{1,\dots,j-1\}}$.

Next, examine:

$$(\star)\begin{cases} X_1 \perp\!\!\!\perp_{k_{\{1,D_x+1\}}} Y_1 \\ X_1 \perp\!\!\!\perp_{k_{\{1,D_x+1,D_x+2\}}} Y_2 \mid Y_1 \\ X_1 \perp\!\!\!\perp_{k_{\{1,D_x+1,D_x+2,D_x+3\}}} Y_3 \mid Y_{\{1,2\}} \\ \qquad\vdots \\ X_1 \perp\!\!\!\perp_{k_{\{1,D_x+1\ldots,D-1\}}} Y_{D_y-1} \mid Y_{\{1,\ldots,D_y-2\}} \\ X_1 \perp\!\!\!\perp_{k_{\{1,D_x+1\ldots,D\}}} Y_{D_y} \mid Y_{\{1,\ldots,D_y-1\}} \end{cases}$$

$$(\star\star)\begin{cases} X_2 \perp\!\!\!\perp_{k_{\{1,2,D_x+1\}}} Y_1 \mid X_1 \\ X_2 \perp\!\!\!\perp_{k_{\{1,2,D_x+1,D_x+2\}}} Y_2 \mid X_1, Y_1 \\ \qquad\vdots \\ X_2 \perp\!\!\!\perp_{k_{\{1,2,D_x+1,\ldots D\}}} Y_{d_y} \mid X_1, Y_{\{1,\ldots,D_y-1\}} \end{cases}$$

$$\vdots$$

$$(\star\star\star)\begin{cases} X_{D_x} \perp\!\!\!\perp_{k_{\{1,\ldots,D_x,D_x+1\}}} Y_1 \mid X_{\{1,\ldots,D_x-1\}} \\ X_{D_x} \perp\!\!\!\perp_{k_{\{1,\ldots,D_x,D_x+1,D_x+2\}}} Y_2 \mid X_{\{1,\ldots,D_x-1\}}, Y_1 \\ \qquad\vdots \\ X_{D_x} \perp\!\!\!\perp_k Y_{D_y} \mid X_{\{1,\ldots,D_x-1\}}, Y_{\{1,\ldots,D_y-1\}} \end{cases}$$

Each of the above rows is obtained by the previous argument. Applying the contraction lemma recursively from top to bottom to each of the rows in $(\star)$ shows that $X_1 \perp\!\!\!\perp_{\{1,D_x,\ldots,D\}} Y$. Further applying the contraction lemma to the latter result and the rows of $(\star\star)$ shows that

$$X_{\{1,2\}} \perp\!\!\!\perp_{\{1,2,D_x,\ldots,D\}} Y.$$

A similar application of the contraction lemma to the previous results and all the rows up to $(\star\star\star)$ shows that $X \perp\!\!\!\perp_k Y$. □

Remark S*1.* For every $i \in \{1,..,D_x\}$ and $j \in \{1,..,D_y\}$, and for each of the above conditions $X_i \perp\!\!\!\perp_{k_{\{1,\ldots,i-1,D_x+1,\ldots,D_x+j\}}} Y_j \mid X_{\{1,\ldots,i-1\}}, Y_{\{1,\ldots,j-1\}}$ there are $(2^{k_i}-1)(2^{k_j}-1)$ one degrees of freedom tests required, each repeated due to the conditioning

$$\prod_{s=1}^{i-1} 2^{k_s} \cdot \prod_{t=D_x+1}^{D-1} 2^{k_t}$$

times. Summing over j, we get that for each i, there are

$$(2^{k_i}-1) \cdot \prod_{s=1}^{i-1} 2^{k_s} \cdot (2^{\sum_{t=D_x+1}^{D} k_t} - 1)$$

one degrees of freedom tests. Summing those over i we get that overall we need to perform

$$(2^{\sum_{s=1}^{D_x} k_s} - 1)(2^{\sum_{t=D_x+1}^{D} k_t} - 1)$$

one degrees of freedom tests.

Under H_1 there are

$$2^{\sum_{s=1}^{D_x} k_s} \cdot 2^{\sum_{t=D_x+1}^{D} k_t} - 1$$

degrees of freedom, under H_0 there are

$$(2^{\sum_{s=1}^{D_x} k_s} - 1) + (2^{\sum_{t=D_x+1}^{D} k_t} - 1)$$

degrees of freedom, and thus we need

$$(2^{\sum_{s=1}^{D_x} k_s} - 1)(2^{\sum_{t=D_x+1}^{D} k_t} - 1)$$

degrees of freedom to identify a difference between the null and the alternative. It follows from the proof that we may indeed use

$$(2^{\sum_{s=1}^{D_x} k_s} - 1)(2^{\sum_{t=D_x+1}^{D} k_t} - 1)$$

1-degree of freedom independence tests to do so.

Proof (Theorem 1). The proof of Theorem 1 is immediate from Lemmas S1 and S6. □

Proof (Theorem 2). Let A be a cuboid in resolution r, $i \in \{1, \ldots, D_x\}$, $j \in \{1, \ldots, D_y\}$ and $p_{ij}(A)$ the p-value that is determined by the table $\{n(A_{ij}^{00}), n(A_{ij}^{01}), n(A_{ij}^{10}), n(A_{ij}^{11})\}$. For any $r > R^*$, whether or not A is selected in the MULTIFIT procedure for testing is determined by the p-values observed on the collection of all potential ancestral cuboids of A. Without loss of generality we let $R^* = 0$ to simplify notation. The general case requires trivial changes to the proof. A cuboid A will be selected in MULTIFIT if there exists a sequence of nested cuboids, or a lineage, $A_0 \subset A_1 \subset \cdots \subset A_r$ of resolution $0, 1, \ldots, r$ respectively such that each A_{k+1} is a child cuboid of A_k in the (i_k, j_k)-face, and moreover, the p-value of the (i_k, j_k)-table of A_k is less than the threshold p^*. As such, the event that a cuboid A is in $\mathcal{C}^{(r)}$ is in the σ-algebra generated by the 2×2-table counts $n(\bar{A}_{ij})$ for all (i, j) pairs and all sets $\bar{A}$ that can be an ancestor cuboid of A along some lineage.

Suppose the resolution-r cuboid A is in the $\mathcal{A}^k$ stratum with

$$|k| = \sum_{d=1}^{D} k_d = r.$$

Also, let

$$r_x = \sum_{d=1}^{D_x} k_d, r_y = \sum_{d=D_x+1}^{D} k_d.$$

Any potential ancestor cuboid of A, denoted by $\bar{A}$, is the union of several sets in $\mathcal{A}^k$, and thus the (i, j)-table of $\bar{A}$, $n(\bar{A}_{ij})$, for every (i, j) pair, is determined exactly if we know the counts in all sets in $\mathcal{A}^k$.

For any positive integer ρ, we denote the collection of all level-ρ marginal partitions of Ω_X as

$$\widetilde{P}_x^{\rho} = \left\{ \mathcal{P}^{k_1} \times \ldots \times \mathcal{P}^{k_{D_x}} : \sum_{d=1}^{D_x} k_d = \rho \right\}$$

and the collection of all level-ρ marginal partitions of Ω_Y as

$$\widetilde{P}_y^{\rho} = \left\{ \mathcal{P}^{k_{D_x+1}} \times \ldots \times \mathcal{P}^{k_D} : \sum_{d=D_x+1}^{D} k_d = \rho \right\}$$

Consider the following sequence of nested marginal partitions on Ω_X, $\widetilde{\mathcal{P}}_x^1 \subset \widetilde{\mathcal{P}}_x^2 \subset \cdots \subset \widetilde{\mathcal{P}}_x^{r_x} \subset \widetilde{\mathcal{P}}_x^{r_x+1} \subset \widetilde{\mathcal{P}}_x^{r_x+2} \subset \cdots \subset \widetilde{\mathcal{P}}_x^{r_x+D_x}$, where $\widetilde{\mathcal{P}}_x^1 \in \widetilde{P}_x^1, \cdots, \widetilde{\mathcal{P}}_x^{r_x+D_x} \in \widetilde{P}_x^{r_x+D_x}$, such that we first divide Ω_{X_1} k_1 times to get $\widetilde{\mathcal{P}}_x^1, \ldots, \widetilde{\mathcal{P}}_x^{k_1}$, followed by dividing Ω_{X_2} k_2 times to get $\widetilde{\mathcal{P}}_x^{k_1+1}, \ldots, \widetilde{\mathcal{P}}_x^{k_1+k_2}$, and so on an so forth until dividing $\Omega_{X_{D_x}}$ k_{D_x} times to get $\widetilde{\mathcal{P}}_x^{r_x-k_{D_x}+1}, \ldots, \widetilde{\mathcal{P}}_x^{r_x}$. Then divide Ω_{X_i} once to get $\widetilde{\mathcal{P}}_x^{r_x+1}$, and finally divide each of the other $D_x - 1$ dimensions once in any order to get $\widetilde{\mathcal{P}}_x^{r_x+2}, \ldots, \widetilde{\mathcal{P}}_x^{r_x+D_x}$.

In exactly the same manner, we can construct a sequence of nested marginal partitions of Ω_Y, $\widetilde{\mathcal{P}}_y^1 \subset \widetilde{\mathcal{P}}_y^2 \subset \cdots \subset \widetilde{\mathcal{P}}_y^{r_y} \subset \widetilde{\mathcal{P}}_y^{r_y+1} \subset \widetilde{\mathcal{P}}_y^{r_y+2} \subset \cdots \subset \widetilde{\mathcal{P}}_x^{r_y+D_y}$, where $\widetilde{\mathcal{P}}_y^1 \in \widetilde{P}_y^1, \cdots, \widetilde{\mathcal{P}}_y^{r_y+D_y} \in \widetilde{P}_y^{r_y+D_y}$, such that we first divide Ω_{Y_1} k_{D_x+1} times to get $\widetilde{\mathcal{P}}_y^1, \ldots, \widetilde{\mathcal{P}}_y^{k_{D_x+1}}$, followed by dividing Ω_{Y_2} k_{D_x+2} times to get $\widetilde{\mathcal{P}}_y^{k_{D_x+1}+1}, \ldots, \widetilde{\mathcal{P}}_y^{k_{D_x+1}+k_{D_x+2}}$, and so on an so forth until dividing $\Omega_{Y_{D_y}}$ k_D times to get $\widetilde{\mathcal{P}}_y^{r_y-k_D+1}, \ldots, \widetilde{\mathcal{P}}_y^{r_y}$. Then divide Ω_{Y_j} once to get $\widetilde{\mathcal{P}}_y^{r_y+1}$, and finally divide each of the other $D_y - 1$ dimensions once in any order to get $\widetilde{\mathcal{P}}_y^{r_x+1}, \ldots, \widetilde{\mathcal{P}}_x^{r_x+D_y}$.

Under these two marginal partition sequences, we have $A \in \widetilde{\mathcal{P}}_x^{r_x} \times \widetilde{\mathcal{P}}_y^{r_y} = \mathcal{A}^k$, whereas the four child cuboids of A with respect to the (i,j)-face are in the two strata $\widetilde{\mathcal{P}}_x^{r_x+1} \times \widetilde{\mathcal{P}}_y^{r_y}$ and $\widetilde{\mathcal{P}}_x^{r_x} \times \widetilde{\mathcal{P}}_y^{r_y+1}$. Moreover, any (i,j)-face of any ancestral cuboid of A are formed by unions of sets that are not in the strata $\widetilde{\mathcal{P}}_x^{r_x+i'} \times \widetilde{\mathcal{P}}_y^{r_y+j'}$ for $i' = 1, 2, \ldots, D_x$ and $j' = 1, 2, \ldots, D_y$.

Now by Theorem 3 in Ma and Mao (2019), conditional on the X and Y marginal values of the observations, the counts in any (i,j)-table of A given the corresponding row and column totals are independent of the σ-algebra generated by all counts in the sets that are not of the form $\widetilde{\mathcal{P}}_x^{r_x+i'} \times \widetilde{\mathcal{P}}_y^{r_y+j'}$ for $i' = 1, 2, \ldots, D_x$ and $j' = 1, 2, \ldots, D_y$. Therefore, the counts in any (i,j)-table of A are also independent of the σ-algebra generated by the 2×2-table counts $n(\bar{A}_{ij})$ for all sets $\bar{A}$ that can be ancestor cuboids of A along some lineage and all (i,j) pairs, and hence are also independent of the selection under the MULTIFIT procedure. This completes the proof. □

Proof (Corollary 1). This corollary follows immediately since the p-value on the (i,j)-table of a cuboid A is determined from the central hypergeometric distribution given the row and column totals of that table, which due to Theorem 2, is the actual sampling distribution of the table given the row and column totals under the null hypothesis of independence whether or not one conditions on the event that A is selected for testing in the MULTIFIT procedure. □

Proof (Theorem 3). We adopt here a similar strategy to what was used in the proof of Theorem 5 in Ma and Mao (2019). We recall that the null distribution of the 2×2 contingency table on a cuboid A arising from an i.i.d. sample conditional on its margins is the central hypergeometric distribution, as discussed in Section 2.3, and the same table when the null does not hold is distributed according to the noncentral hypergeometric distribution. Zhou (2019) developed a normal approximation for the noncentral hypergeometric distribution and accompanying results on which we rely.

Under the conditions in the theorem's statement, we can take $R_{max} = R^*$, that is, exhaustive testing. Then according to our Strategy 1 for multiple testing adjustment, or the holistic approach with Bonferroni's correction

$$\alpha_{ij}(A) = \alpha / \left(\sum_{\rho=0}^{R_{max}} D_x \cdot D_y \cdot 2^{\rho} \cdot \binom{\rho + D - 1}{D - 1} \right)$$

is the table-specific threshold. According to our Strategy 2 for multiple testing adjustment, or the resolution-specific approach with exhaustive testing up to resolution R_{max} and utilizing Bonfer-

roni's correction within resolution as well as between resolutions

$$\alpha_{ij}(A) = \alpha / \left(D_x \cdot D_y \cdot 2^r \cdot \binom{r + D - 1}{D - 1} \cdot R_{max} \right)$$

is the table-specific threshold. The selection of Bonferroni's correction here ensures that the proof is valid for any correction that is less conservative, e.g. Holm's correction that we use in our implementation.

We start by proving the theorem in a simplified case where we assume that $R^* = R_{max}$ is fixed but large enough such that $r \leq R^*$. Under both instances $\alpha_{ij}(A)$ is constant when R^* is constant. Let then $\theta_{ij}(A) = \theta$. Without loss of generality, let us assume that $\theta > 1$.

By Theorem 2.2 in Zhou (2019), we have that given $n(A_{ij}^{0\cdot}), n(A_{ij}^{\cdot 0}), n(A)$,

$$Z_{n,\theta,i,j}(A) = \frac{n(A_{ij}^{00}) - \mathrm{E}_\theta\{n(A_{ij}^{00}) \mid n(A_{ij}^{0\cdot}), n(A_{ij}^{\cdot 0}), n(A)\}}{\mathrm{var}_\theta^{1/2}\{n(A_{ij}^{00}) \mid n(A_{ij}^{0\cdot}), n(A_{ij}^{\cdot 0}), n(A)\}} \to_{\mathcal{L}} \mathrm{N}(0,1).$$

$p_{ij}(A)$, the p-value for the table determined by $n(A_{ij}^{00})$ when the margins $\{n(A_{ij}^{0\cdot}), n(A_{ij}^{\cdot 0}), n(A)\}$ are given, is computed for the two sided version of Fisher's exact test by summing the probabilities of all tables that are more extreme than the given one. That is, those with a smaller probability of occurrence compared to the table for which the p-value is computed according to the central hypergeometric distribution.

We can utilize the form $Z_{n,\theta,i,j}$ to write the probability of rejection for the test on the (i,j)-table of A. If $n(A_{ij}^{00})$ is less than or equal to the mode of the hypergeometric distribution with the parameters $\{n(A_{ij}^{0\cdot}), n(A_{ij}^{\cdot 0}), n(A)\}$ we have

$$\begin{aligned} &\mathrm{pr}\{p_{ij}(A) < \alpha_{ij}(A) \mid \theta_{ij}(A) = \theta, n_{r_x,0}, n_{0,r_y}\} \\ &> \mathrm{pr}\left[Z_{n,1,i,j}(A) > F_{A,n}^{-1}\{\alpha_{ij}(A)\} \mid \theta_{ij}(A) = \theta, n_{r_x,0}, n_{0,r_y} \right] \end{aligned}$$

where $F_{A,n}$ denotes the exact cdf of $Z_{n,1,i,j}$ given the marginal totals. Else, if $n(A_{ij}^{00})$ is greater than the mode of the hypergeometric distribution with the parameters $\{n(A_{ij}^{0\cdot}), n(A_{ij}^{\cdot 0}), n(A)\}$

$$\begin{aligned} &\mathrm{pr}\{p_{ij}(A) < \alpha_{ij}(A) \mid \theta_{ij}(A) = \theta, n_{r_x,0}, n_{0,r_y}\} \\ &> \mathrm{pr}\left[Z_{n,1,i,j}(A) > F_{A,n}^{-1}\{1 - \alpha_{ij}(A)\} \mid \theta_{ij}(A) = \theta, n_{r_x,0}, n_{0,r_y} \right]. \end{aligned}$$

Without loss of generality we continue to work with the second case.

Assume now that indeed $\theta_{ij}(A) = \theta \neq 1$. Then

$$\begin{aligned} &\lim_{n\to\infty} \mathrm{pr}\left[Z_{n,1,i,j}(A) > F_{A,n}^{-1}\{1 - \alpha_{ij}(A)\} \mid \theta_{ij}(A) = \theta, n_{r_x,0}, n_{0,r_y} \right] \\ &= \lim_{n\to\infty} \mathrm{pr}\left[Z_{n,\theta,i,j}(A) > c_n F_{A,n}^{-1}\{1 - \alpha_{ij}(A)\} - c_n d_n \,\middle|\, \theta_{ij}(A) = \theta, n_{r_x,0}, n_{0,r_y} \right] \end{aligned}$$

where

$$c_n = \frac{\mathrm{var}_1^{1/2}\{n(A_{ij}^{00}) \mid n(A_{ij}^{0\cdot}), n(A_{ij}^{\cdot 0}), n(A)\}}{\mathrm{var}_\theta^{1/2}\{n(A_{ij}^{00}) \mid n(A_{ij}^{0\cdot}), n(A_{ij}^{\cdot 0}), n(A)\}}$$

and

$$d_n = \frac{\mathrm{E}_\theta\{n(A_{ij}^{00}) \mid n(A_{ij}^{0\cdot}), n(A_{ij}^{\cdot 0}), n(A)\} - \mathrm{E}_1\{n(A_{ij}^{00}) \mid n(A_{ij}^{0\cdot}), n(A_{ij}^{\cdot 0}), n(A)\}}{\mathrm{var}_1^{1/2}\{n(A_{ij}^{00}) \mid n(A_{ij}^{0\cdot}), n(A_{ij}^{\cdot 0}), n(A)\}}.$$

By Corollary 2.1 in Zhou (2019) we have $1/\sqrt{\max(\theta,\theta^{-1})} \leq c_n \leq 1/\sqrt{\min(\theta,\theta^{-1})}$ for all n and $d_n \asymp \sqrt{n}$ with F^∞ probability 1.

By the above normal approximation $F_{A,n}^{-1}\{1-\alpha_{ij}(A)\} \to \Phi^{-1}\{1-\alpha_{ij}(A)\}$. Therefore $c_n\Phi^{-1}\{1-\alpha_{ij}(A)\} - c_n d_n \to -\infty$ with F^∞ probability 1, and so with F^∞ probability 1,

$$\lim_{n\to\infty} \mathrm{pr}\{p_{ij}(A) < \alpha_{ij}(A) \mid n_{r_x,0}, n_{0,r_y}\} = 1.$$

This completes the proof for the case where R^* is constant since the null of independence is rejected whenever $p_{ij}(A) < \alpha_{ij}(A)$ for some A, i and j.

Assume now that $R_{max} = R^* = o(\log n)$. Under both the holistic and resolution-specific strategies for multiple testing we have $\sqrt{n}\cdot\alpha_{ij,n}(A) \to \infty$ as $n \to \infty$.

Since $c_n d_n = O(\sqrt{n})$ with F^∞ probability 1, we need to show that $c_n F_{A,n}^{-1}\{1-\alpha_{ij}(A)\} = o(\sqrt{n})$ with F^∞ probability 1 in order to establish consistency. Rewrite:

$$c_n F_{A,n}^{-1}\{1-\alpha_{ij,n}(A)\} = \\ c_n\left[F_{A,n}^{-1}\{1-\alpha_{ij,n}(A)\} - \Phi^{-1}\{1-\alpha_{ij,n}(A)\}\right] + c_n\Phi^{-1}\{1-\alpha_{ij,n}(A)\}$$

Examine $c_n[F_{A,n}^{-1}\{1-\alpha_{ij,n}(A)\} - \Phi^{-1}\{1-\alpha_{ij,n}(A)\}]$: by Theorem 2.3 of Zhou (2019), we have with F^∞ probability 1,

$$|\Phi[F_{A,n}^{-1}\{1-\alpha_{ij,n}(A)\}] - \{1-\alpha_{ij,n}(A)\}| < \gamma/\sqrt{n}$$

for some positive constant γ and large enough n. Therefore

$$\Phi^{-1}\{1-\alpha_{ij,n}(A) - \gamma/\sqrt{n}\} < F_{A,n}^{-1}\{1-\alpha_{ij,n}(A)\} < \Phi^{-1}\{1-\alpha_{ij,n}(A) + \gamma/\sqrt{n}\}.$$

Since $1-\Phi(x) \asymp e^{-x^2/2}/x$ as $x \to \infty$ and $\sqrt{n}\cdot\alpha_{ij,n}(A) \to \infty$ as $n \to \infty$, we have

$$|\Phi^{-1}\{1-\alpha_{ij,n}(A) - \gamma/\sqrt{n}\} - \Phi^{-1}\{1-\alpha_{ij,n}(A)\}| \to 0$$

and

$$|\Phi^{-1}\{1-\alpha_{ij,n}(A) + \gamma/\sqrt{n}\} - \Phi^{-1}\{1-\alpha_{ij,n}(A)\}| \to 0$$

Hence,

$$|F_{A,n}^{-1}\{1-\alpha_{ij,n}(A)\} - \Phi^{-1}\{1-\alpha_{ij,n}(A)\}| \to 0.$$

Examine $c_n\Phi^{-1}\{1-\alpha_{ij,n}(A)\}$: since $1-\Phi(x) \asymp e^{-x^2/2}/x$ as $x \to \infty$ and $\sqrt{n}\cdot\alpha_{ij,n}(A) \to \infty$ as $n \to \infty$, we have $\Phi^{-1}\{1-\alpha_{ij,n}(A)\} = o(\sqrt{n})$. Therefore, we have with F^∞ probability 1 that $c_n F_{A,n}^{-1}\{1-\alpha_{ij}(A)\} = o(\sqrt{n})$. That is, we have with probability 1 $p_{ij}(A) < \alpha_{ij}(A)$ for some A, i and j and therefore the null of independence is rejected with probability 1. □

2. PSEUDO-CODE FOR THE MULTIFIT PROCEDURE

Algorithm S*1.* MULTIFIT procedure for testing multivariate independence.

Let $\mathcal{C}^{(r)}$ be the collection of all cuboids of resolution r for $r = 0, 1, \dots, R^*$,
and let $\mathcal{C}^{(r)} = \emptyset$ for $r = R^* + 1, \dots, R_{max}$.
For $r = 0$ to $r = R_{max}$
    For each $A \in \mathcal{C}^{(r)}$
        For $i = 1$ to $i = D_x$
            For $j = 1$ to $j = D_y$
                Apply Fisher's exact test on the (i, j)-table
                of A and record the p-value
                If $R^* \leq r < R_{max}$
                    If the (i, j)-table of A has a p-value smaller than
                    a threshold p^*
                        Add the four half cuboids of A into $\mathcal{C}^{(r+1)}$
Output the list of p-values
Apply multiple testing adjustment on these p-values.

3. SCENARIOS FOR THE POWER STUDY

Table S1. *Simulation Scenarios*

Scenario	# of Data Points	Max Res	Simulation Setting
Sine	300	4	$X_1 = Z, Y_1 = Z', X_2 = U,$<br>$Y_2 = \sin(5\pi X_2) + 4\epsilon$
Circular	300	4	$X_1 = Z, Y_1 = Z', \theta \sim \text{Uniform}(-\pi, \pi)$<br>$X_2 = \cos(\theta) + \epsilon, Y_2 = \sin(\theta) + \epsilon'$
Checkerboard	1500	5	$W \sim$ Multi-Bern$(\{1,2,3,4,5\}, (1/5,1/5,1/5,1/5,1/5))$<br>$V_1 \sim$ Multi-Bern$(\{1,3,5\}, (1/3,1/3,1/3))$<br>$V_2 \sim$ Multi-Bern$(\{2,4\}, (1/2,1/2))$<br>$X_1 = Z, Y_1 = Z', X_2 = W + \epsilon,$<br>$Y_2 = \begin{cases} V_1 + \epsilon', & \text{if } W \text{ is odd} \\ V_2 + \epsilon', & \text{if } W \text{ is even} \end{cases}$
Linear	300	4	$X_1 = Z, Y_1 = Z', X_2 = U,$<br>$Y_2 = X_2 + 3\epsilon$
Parabolic	300	4	$X_1 = Z, Y_1 = Z', X_2 = U,$<br>$Y_2 = (X_2 - 0.5)^2 + 0.75\epsilon$
Local	1000	6	$X_1 = Z, Y_1 = Z', X_2 = Z'',$<br>$Y_2 = \begin{cases} X_2 + \epsilon/6 & \text{if } 0 < X_2, Z''' < 0.7 \\ Z''', & \text{otherwise} \end{cases}$

Six simulation scenarios. In all cases, Z, Z', Z'', Z''' are i.i.d $\mathrm{N}(0,1)$. At each noise level $l = 1, 2, ..., 20$, ϵ, ϵ' and ϵ'' are i.i.d $\mathrm{N}\{0, (l/20)^2\}$, and $U \sim \mathrm{Uniform}(0,1)$. The maximal resolution is the algorithm's default: $\lfloor \log_2(n/10) \rfloor$ where n is the number of data points.

Table S2. *'Spread' Simulation Scenarios (part 1)*

Scenario	# of Data Points	Max Res	Simulation Setting
Sine	300	4	$X_1 = U, X_2 = U$<br>$Y_1 = \sin(5\pi X_1) + \cos(5\pi X_2) + 4\epsilon$<br>$Y_2 = -\sin(5\pi X_2) + \cos(5\pi X_1) + 4\epsilon'$
Circular	300	4	$Z_1, Z_2 \sim Beta(0.9, 0.27)$<br>$b_1, b_2, b_3, b_4 \sim$ Multi-Bern$(\{-1, 1\}, (0.5, 0.5))$<br>$X_1 = b_1 Z_1 + \epsilon$, $X_2 = b_2 Z_2 + \epsilon'$<br>$Y_1 = b_3\{1 - (0.2b_1 Z_1 + 0.8b_2 Z_2)^2\}^{1/2} + \epsilon''$<br>$Y_2 = b_4\{1 - (0.8b_1 Z_1 + 0.2b_2 Z_2)^2\}^{1/2} + \epsilon'''$
Checkerboard	1500	5	$W, W' \sim$ Multi-Bern$(\{1, 2, 3, 4, 5\}, (0.2, 0.2, 0.2, 0.2, 0.2))$<br>$X_1 = W + 0.75^2\epsilon, X_2 = W' + 0.75^2\epsilon'$<br>$m_1 \sim$ Multi-Bern$(\{1, 2\}, (0.2, 0.8))$<br>$m_2 \sim$ Multi-Bern$(\{1, 2\}, (0.8, 0.2))$<br>$V_1, V_1' \sim$ Multi-Bern$(\{1, 3, 5\}, (1/3, 1/3, 1/3))$<br>$V_2, V_2' \sim$ Multi-Bern$(\{2, 4\}, (0.5, 0.5))$<br>$Y_1 = \begin{cases} V_1 + 0.75^2\epsilon'', & \text{if } x_{m_1} \text{ is odd} \\ V_2 + 0.75^2\epsilon'', & \text{if } x_{m_1} \text{ is even} \end{cases}$<br>$Y_2 = \begin{cases} V_1' + 0.75^2\epsilon''', & \text{if } x_{m_2} \text{ is odd} \\ V_2' + 0.75^2\epsilon''', & \text{if } x_{m_2} \text{ is even} \end{cases}$

$\epsilon, \epsilon', \epsilon''$ and ϵ''' are i.i.d $\mathrm{N}\{0, (l/20)^2\}$, and $U \sim \mathrm{Uniform}(0, 1)$. The maximal resolution is the algorithm's default: $\lfloor \log_2(n/10) \rfloor$ where n is the number of data points.

Table S3. *'Spread' Simulation Scenarios (part 2)*

Scenario	# of Data Points	Max Res	Simulation Setting
Linear	300	4	$X_1, X_2 \sim U(0,1)$<br>$Y_1 = X_1 - 2X_2 + 6\epsilon$, $Y_2 = -X_1 + X_2 + 6\epsilon'$
Parabolic	300	4	$X_1, X_2 \sim \mathrm{Uniform}(0,1)$<br>$Y_1 = (X_1 - 0.5)^2 - (X_2 - 0.5)^2 + 0.75\epsilon$<br>$Y_2 = (X_2 - 0.5)^2 - (X_1 - 0.5)^2 + 0.75\epsilon'$
Local	1000	6	A spread linear signal scaled to (0,0.7) for all margins:<br>$x_1, x_2 \sim U(0, 0.7)$<br>$y_1 = 0.7(x_1 - x_2 + 1.4)/2.1 + 1/6 \cdot \epsilon$<br>$y_2 = 0.7(-x_1 + x_2 + 0.7)/1.4 + 1/6 \cdot \epsilon'$<br>Embedded within a small portion of the space:<br>$X_1 = Z, X_2 = Z'$<br>$Y_1 = \begin{cases} y_1 & \text{if } 0 < X_1, Z'' < 0.7 \\ Z'', & \text{otherwise} \end{cases}$<br>$Y_2 = \begin{cases} y_3 & \text{if } 0 < X_2, Z''' < 0.7 \\ Z'', & \text{otherwise} \end{cases}$

Z, Z', Z'', Z''' are i.i.d $\mathrm{N}(0,1)$. At each noise level $l = 1, 2, ..., 20$, ϵ, ϵ' and ϵ'' are i.i.d $\mathrm{N}\{0, (l/20)^2\}$, and $U \sim \mathrm{Uniform}(0,1)$. The maximal resolution is the algorithm's default: $\lfloor \log_2(n/10) \rfloor$ where n is the number of data points.

4. NUMERICAL VALIDATION OF LEVEL CONTROL THROUGH SIMULATIONS

To demonstrate that the multiscale Fisher's independence test properly controls the level we executed 500 simulations with the default tuning parameters for various sample sizes. The underlying data $\{X_{1i}\}$, $\{X_{2i}\}$, $\{Y_{1i}\}$ and $\{Y_{2i}\}$ are drawn independently from a standard normal distribution for $i \in \{1, \ldots, n\}$ with $n \in \{100, 200, \ldots, 2000\}$. Figure S4 shows the estimated level for the multiscale Fisher's independence test with different variations for the independence tests on each table and multiple testing adjustment options on Fisher's exact test with mid-p corrected p-values. See Agresti and Gottard (2007) for a discussion on the mid-p correction.

The results confirm the theoretical guarantees that the level can be controlled at any given level α. In fact, the procedure appears to be a bit conservative in controlling the level. Note, although X and Y are independent under the null hypothesis, the dependency structure between the different margins of X is arbitrary, as well as the dependency structure between the different margins of Y. Therefore, this simulation is not exhaustive. However, we repeated the estimation of the level under various dependency structures for the margins, and the results are consistent with these that are shown here.

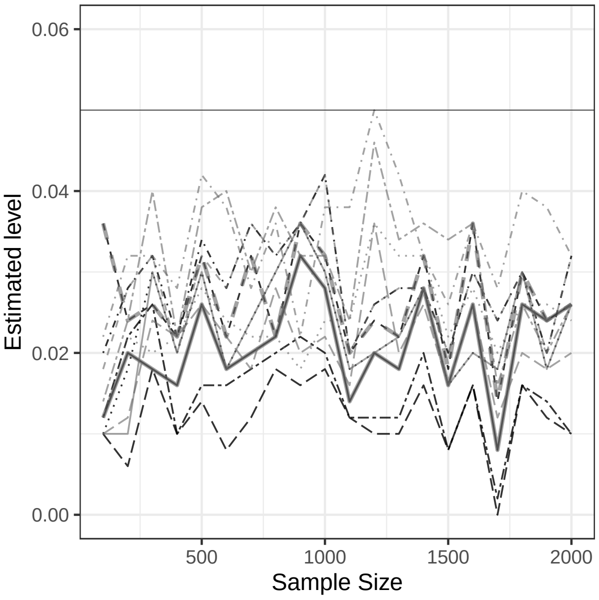

Fig. S4. Estimated level for MultiFIT versus sample size. Fourteen different variants of the method are examined: the holistic strategy with Holm's correction (black solid), the holistic strategy with Holm's and mid-p correction (black dashes), the resolution specific strategy with Holm's correction (black dotted), the resolution specific strategy with Holm's and mid-p correction (black dot-dash), the early stopping strategy with Holm's correction (black long-dashes), the early stopping strategy with Holm's and mid-p correction (black two-dash), the holistic strategy with the approximation keeps up to 10 most significant p-values at each resolution and Holm's correction (thick grey solid), the holistic strategy with the approximation keeps up to 10 most significant p-values at each resolution and Holm's and mid-p correction (thick grey dashes), the resolution specific strategy with the approximation keeps up to 10 most significant p-values at each resolution and Holm's correction (grey solid), the resolution specific strategy with the approximation keeps up to 10 most significant p-values at each resolution and Holm's and mid-p correction (grey dashes), the holistic strategy with the approximation keeps up to 100 most significant p-values at each resolution and Holm's correction (grey dotted), the holistic strategy with the approximation keeps up to 100 most significant p-values at each resolution and Holm's and mid-p correction (grey dot-dashe), the resolution specific strategy with the approximation keeps up to 100 most significant p-values at each resolution and Holm's correction (grey long dashes), the resolution specific strategy with the approximation keeps up to 100 most significant p-values at each resolution and Holm's and mid-p correction (grey two dashes).

5. SCALING: COMPARISON OF SCENARIOS FOR THE MULSTISCALE FISHER'S INDEPENDENCE TEST

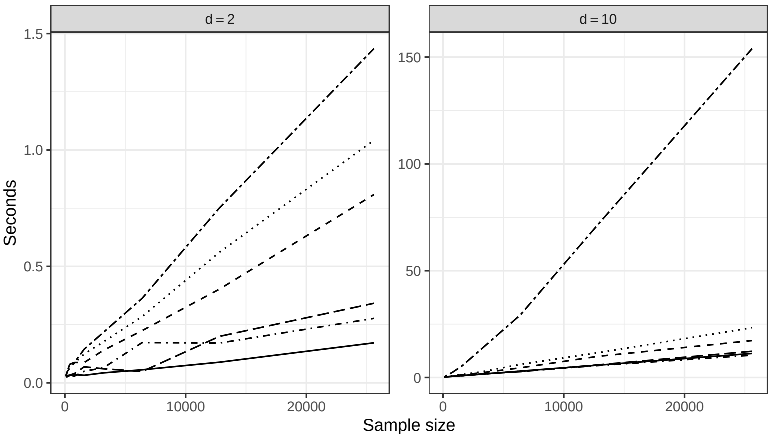

Fig. S5. Computational scalability: run-time vs. sample size for the six simulation scenarios from Table S1 when fitted with the multiscale Fisher's independence test in different dimensionalities with $D_x = D_y = d$, $R^* = 1$ and $l = 3$. 'Null' scenario (solid), 'parabolic' (dashes), 'sine' (dotted), 'checkerboard' (dot-dash), 'circle' (long dashes) and 'local' (two-dash). In all cases the 'linear' scenario requires the most computations and the 'null' scenario the least.

6. SCALING: COMPARISON OF THE MULSTISCALE FISHER'S INDEPENDENCE TEST TO OTHER NEAR LINEAR TIME TESTS

Figure S6 shows a comparison between the multiscale Fisher's independence test and the kernel partial correlation coefficient methods from Deb et al. (2020).

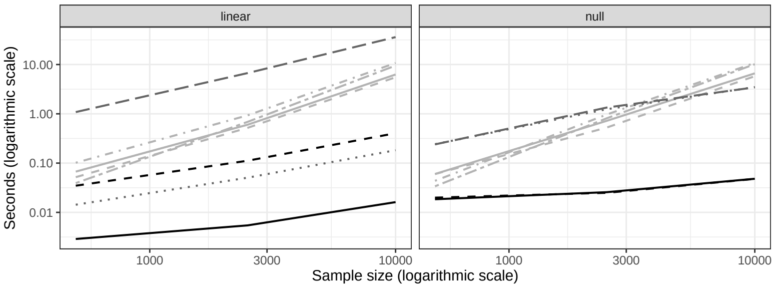

Fig. S6. Computational scalability: run-time vs. sample size. Left panel: 'linear' scenario; Right panel: 'null' scenario. Two variants of MULTIFIT were tested: full method on data with $D_x = D_y = 2$ (black solid) and on data with $D_x = D_y = 10$ (dark grey long dashes), MultiFIT with early stopping on data with $D_x = D_y = 2$ (black dashes) and on data with $D_x = D_y = 10$ (black dotted); MULTIFIT was run with $R^* = 1$ and $p^* = \{D_x D_y \log_2(n)\}^{-1}$. Two variants of the kernel partial correlation method were computed: the unconditional version of graph-based kernel partial correlation using directed K-NN graph on data with $D_x = D_y = 2$ (grey dashes) and on data with $D_x = D_y = 10$ (grey solid); the unconditional version of graph-based kernel partial correlation using directed K-NN graph on data with $D_x = D_y = 2$ (grey dot-dash) and on data with $D_x = D_y = 10$ (grey two-dash). The multiscale Fisher's independence test does not require permutations. The kernel partial correlation method requires permutations for level control and the reported time is for a single permutation.

7. SENSITIVITY ANALYSIS FOR THE PARAMETERS p^* AND R^* OF THE MULTISCALE FISHER'S INDEPENDENCE TEST

In Fig. S7 and Fig. S8 we demonstrate the effect of our main tuning parameters, p^* and R^*, on the estimated power: $R^* = 1$ (solid), $R^* = 2$ (dashes), $R^* = 3$ (dotted), $R^* = 4$ (dot-dash). We evaluate the power for the simulation scenarios that are detailed in Table S1 over the grid $p^* \times R^*$ where $p^* = \{0.005, 0.01, 0.05, 0.1, 0.5\}$ and $R^* = \{1, 2, 3, 4\}$. In general, higher p^* and higher R^* values entail more testing. Under some scenarios, where the dependency structure is 'more local', higher values are needed to ensure better power. When the dependency structures are 'more global' we see that the selection of the tuning parameters makes little difference to the estimated power.

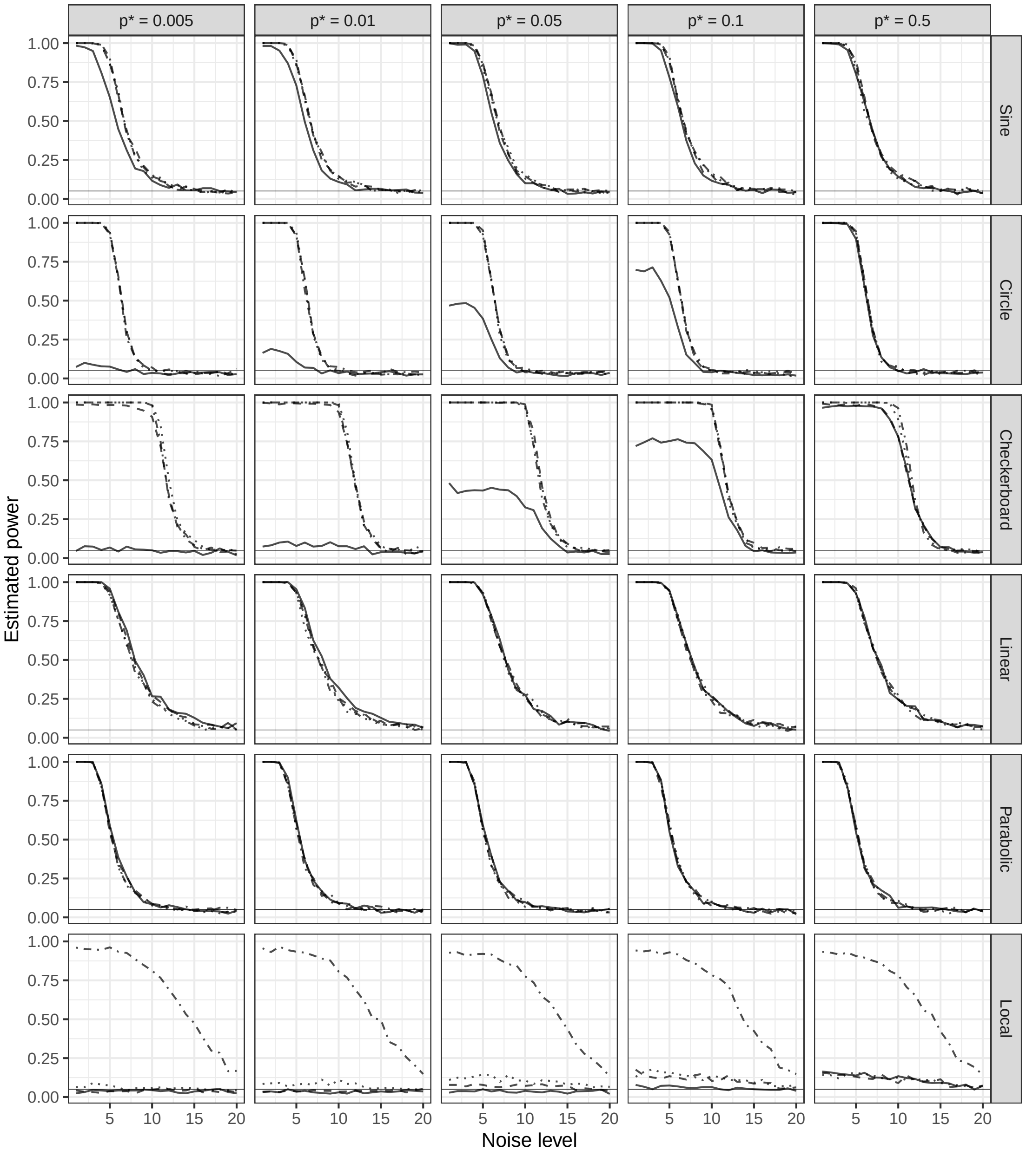

Fig. S7. Power versus noise level for different specifications of MultiFIT. Estimated power at 20 noise levels for the multiscale Fisher's independence test with different p^* and R^* values under the six scenarios from Table S1

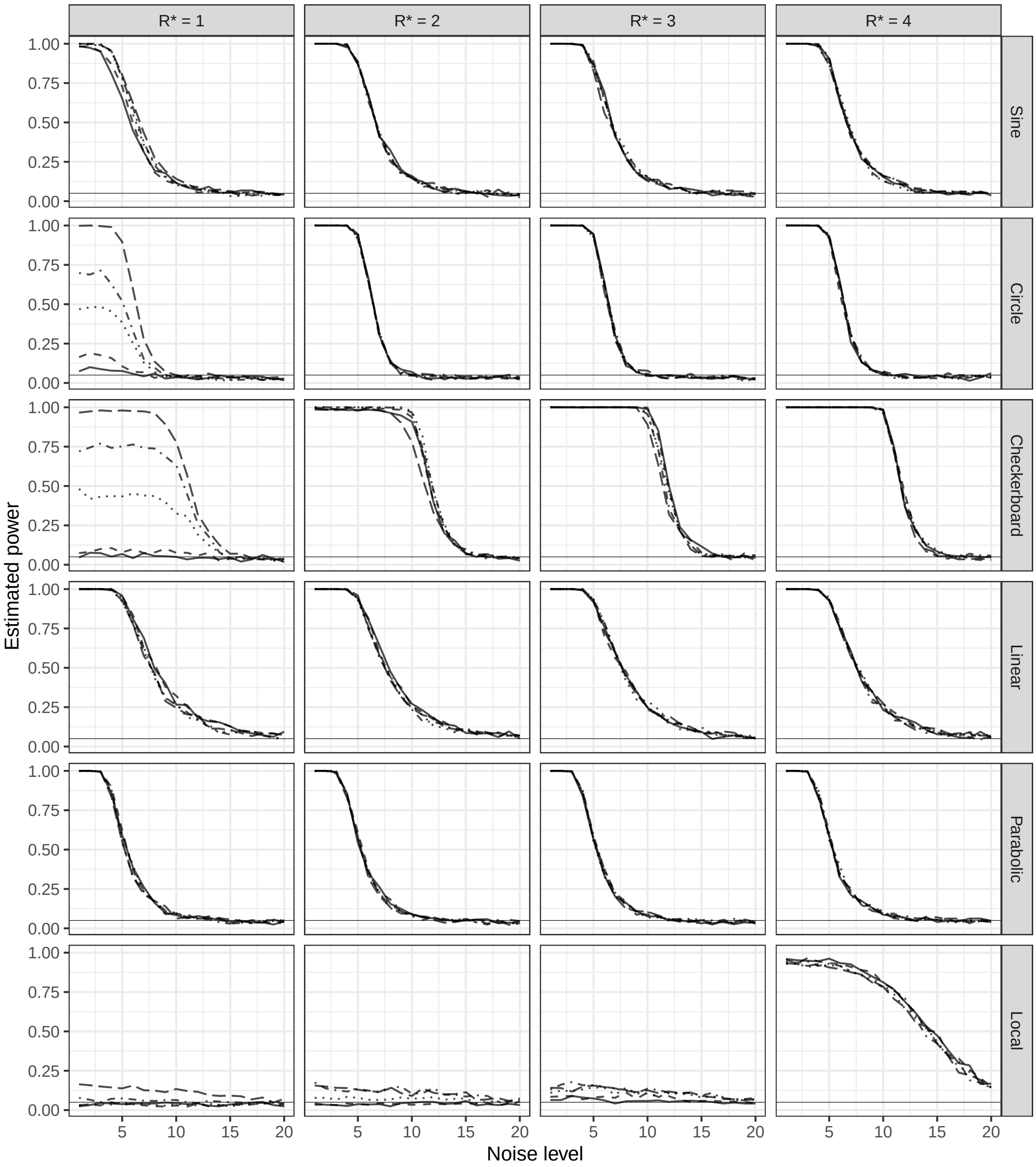

Fig. S8. Power versus noise level for different specifications of MultiFIT. $p^* = 0.005$ (solid), $p^* = 0.01$ (dashes), $p^* = 0.05$ (dotted), $p^* = 0.1$ (dot-dash), $p^* = 0.5$ (long dashes). Estimated power at 20 noise levels for the multiscale Fisher's independence test with different p^* and R^* values under the six scenarios from Table S1

8. SOFTWARE

For the MULTIFIT procedure we used our R package `MultiFit` on the comprehensive R archive network, or CRAN. For the Heller-Heller-Gorfine test (Heller et al., 2013) we used the `HHG` package on CRAN. For distance covariance (Székely and Rizzo, 2009) we used the `energy` package on CRAN. For Brownian distance covariance (Pfister et al., 2018) we used the `dHSIC` package on CRAN.

REFERENCES

Agresti, A. and Gottard, A. (2007). Nonconservative exact small-sample inference for discrete data. *Computational Statistics & Data Analysis*, 51(12):6447 – 6458.

Deb, N., Ghosal, P., and Sen, B. (2020). Measuring association on topological spaces using kernels and geometric graphs.

Heller, R., Heller, Y., and Gorfine, M. (2013). A consistent multivariate test of association based on ranks of distances. *Biometrika*, 100:503–510.

Ma, L. and Mao, J. (2019). Fisher exact scanning for dependency. *Journal of the American Statistical Association*, 114(525):245–258.

Pfister, N., Bühlmann, P., Schölkopf, B., and Peters, J. (2018). Kernel-based tests for joint independence. *Journal of the Royal Statistical Society: Series B (Statistical Methodology)*, 80(1):5–31.

Székely, G. J. and Rizzo, M. L. (2009). Brownian distance covariance. *The Annals of Applied Statistics*, 3(4):1236–1265.

Zhou, Q. (2019). Asymptotics of multivariate contingency tables with fixed marginals. *Journal of Statistical Planning and Inference*, 198:165–170.
